# COVID-19 on the spectrum: a scoping review of hygienic standards

**DOI:** 10.3389/fpubh.2023.1202216

**Published:** 2023-11-01

**Authors:** Chrysa Voidarou, Georgios Rozos, Elisavet Stavropoulou, Elpida Giorgi, Christos Stefanis, Georgios Vakadaris, Natalia Vaou, Christina Tsigalou, Yiannis Kourkoutas, Eugenia Bezirtzoglou

**Affiliations:** ^1^School of Agriculture, University of Ioannina, Arta, Greece; ^2^Veterinary Directorate, South Aegean Region, Ermoupolis, Greece; ^3^Department of Medicine, Lausanne University Hospital (CHUV), University of Lausanne, Lausanne, Switzerland; ^4^Laboratory of Hygiene and Environmental Protection, Department of Medicine, Democritus University of Thrace, Alexandroupolis, Greece; ^5^Laboratory of Hygiene and Environmental Protection, Medical School, Democritus University of Thrace, Alexandroupolis, Greece; ^6^Laboratory of Applied Microbiology and Biotechnology, Department of Molecular Biology and Genetics, Democritus University of Thrace, Alexandroupolis, Greece

**Keywords:** COVID-19, hand hygiene, air, orofecal transmission, food, droplets size

## Abstract

The emergence of COVID-19 in Wuhan, China, rapidly escalated into a worldwide public health crisis. Despite numerous clinical treatment endeavors, initial defenses against the virus primarily relied on hygiene practices like mask-wearing, meticulous hand hygiene (using soap or antiseptic solutions), and maintaining social distancing. Even with the subsequent advent of vaccines and the commencement of mass vaccination campaigns, these hygiene measures persistently remain in effect, aiming to curb virus transmission until the achievement of herd immunity. In this scoping review, we delve into the effectiveness of these measures and the diverse transmission pathways, focusing on the intricate interplay within the food network. Furthermore, we explore the virus's pathophysiology, considering its survival on droplets of varying sizes, each endowed with distinct aerodynamic attributes that influence disease dispersion dynamics. While respiratory transmission remains the predominant route, the potential for oral-fecal transmission should not be disregarded, given the protracted presence of viral RNA in patients' feces after the infection period. Addressing concerns about food as a potential viral vector, uncertainties shroud the virus's survivability and potential to contaminate consumers indirectly. Hence, a meticulous and comprehensive hygienic strategy remains paramount in our collective efforts to combat this pandemic.

## 1. Introduction

Viral infections can cause a wide range of illnesses in humans due to the selective ability of viruses to infect different tissues. Human viruses that cause significant global public health problems include human immunodeficiency virus (HIV), causing severe immunodeficiency, and hepatitis B virus (HBV) and hepatitis C virus (HCV), causes liver damage. Viruses that infect the digestive tract (e.g., rotavirus, astroviruses) or the nervous system (e.g., Zika virus; ZKV and West Nile virus; WNV), or lungs (e.g., Influ-enza, respiratory syncytial virus; RSV) are associated with digestive, neurological and pulmonary symptoms respectively. Furthermore, viruses such as the human T-cell lymphotropic virus (HTLV-I), the human papilloma virus (HPV) and the Merkel virus are associated with multiple types of cancer (1, 2). The full spectrum of known viruses that infect human host cells (i.e., the human virome) and their impact on health needs to be completed (3).

Severe Acute Respiratory Syndrome Coronavirus 2 (SARS-CoV-2) is the causal agent of Coronavirus 2019 (COVID-19), a respiratory disease in humans initially diagnosed and described in 2019. SARS-CoV-2 is a coronavirus with distinct characteristics from previous coronaviruses. The dispersion of COVID-19 has been rather aggressive and has occurred over a relatively short period; the virus has been transmitting to the entire globe except perhaps for Antarctica. As a result, WHO classified COVID-19 as a very serious pandemic at 11/03/2020, threatening potentially the lives of millions of people (4, 5).

Spanish Flu outbreak at the ending of WWI/ in the beginning of the 20th century, the previous large-scale pandemic with many millions' dead patients has set an example to humanity. A careful study of various published reports since 1918, shows the realistic and threatening possibility of an emerging infectious disease and addresses that ≪Today's endemic disease was figuratively yesterday's novel disease≫. In addition, most of the emerging infectious diseases that have an impact on or threaten human health originate from wildlife species, in other words are “zoonotic,” that is of animal origin (6, 7). Wild animals serve as reservoir of viruses and when come to close contact with sensitive domestic species transmit the virus to them. It follows that when the domestic species carry a heavy viral load then the transmission of the virus to humans is quite easy and self-evident (8).

The increased international travel (even to exotic destinations), the international trade of certain commodities such as food and feedstuff, the expansion of agricultural lands and the following deforestation and fragmentation of the natural habitats and, of course, the urbanization of the wilderness increased the interface and hence the chance of contact between humans, domestic animals and wild animals. Thus, an increased spillover effect might occur in a way that Amirian (8) describe as an “epidemiological bridge” favoring the approach of the viral agent to the natives (7–10).

**Figure 1:**
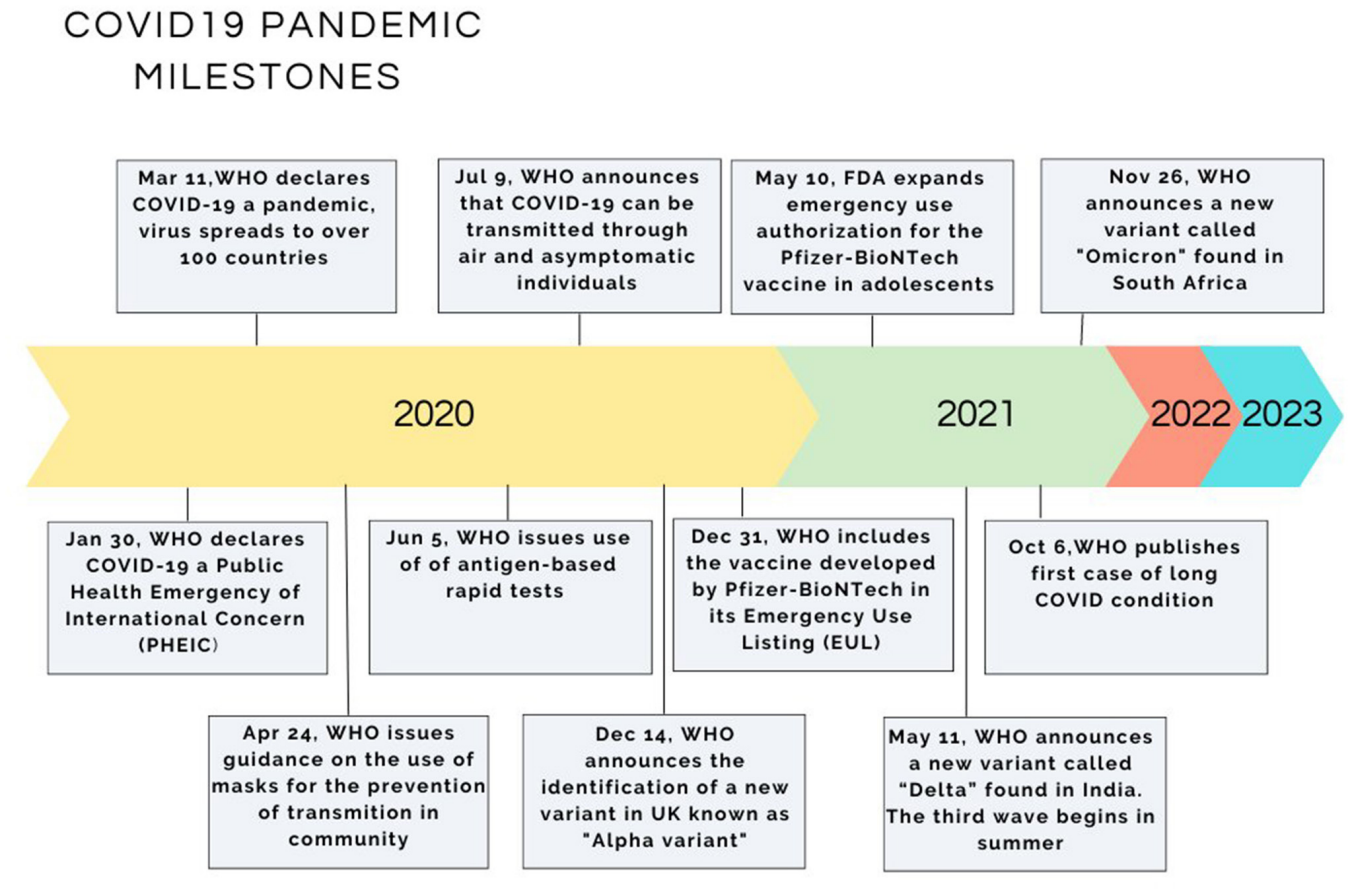
A timeline of the WHO's COVID-19 response and actions taken on a global scale.

In retrospect of the last two decades, several cases where viruses originating from animals have been transmitted to humans have been reported resulting in serious out-breaks. Setting as a time reference the avian influenza (H5N1) outbreaks in 1997–1999 in Hong Kong where the virus was transmitted from poultry to humans, then followed the new coronavirus of the Beta variant (SARS coronavirus CoV) in Guangdong province in China in 2002. The source of the latter outbreak, although not clarified, is thought to be the palm civet cat acting as an animal reservoir (11). 10 years later, in 2012, a new coronavirus emerged in Saudi Arabia (MERS-CoV), originating from bats and using the dromedary camels as intermediate host, causing a respiratory syndrome and affecting a total of 2,494 people, 858 of whom died (fatality rate 34%) (12). Up to very recently the world was facing the new coronavirus pandemic which is believed to originate from bats due to the resemblance with the already known bat virus HKU9-1 (13). This virus passed from bats to civet cats, to pangolins and to other animals sold in the wet markets of China and from them to humans. The WHO declared on 30/01/2020 the disease a Public Health Emergency of International Concern and on 11/03/2020 a pandemic (Figure 1) (4, 5). A thorough examination of the biological world reveals that every cellular life form hosts its viruses or at least virus-like genetic elements. These viruses depend on their host cells for their survival (2). A virus requires a living host cell to replicate (varies accordingly to viruses: humans, animals, plants, and bacteria) (2). For more than 70 years, various species of the coronavirus genus have been isolated from humans and animals. A 1,937 report describes the outbreak of infectious bronchitis in poultry, a disease annihilating the poultry stock, its causative agent being a coronavirus (Coronavirus: Disease Briefings. Cortellis™, a Clarivate Analytics solution, 2020) (14). The prototype murine coronavirus strain (JHM) was isolated in 1949 (15). The first isolation of human coronavirus strains occurred in 1965 from the respiratory tract of patients suffering from the common cold (16). Since the 1970s, the mechanism of replication at a molecular level and the pathogenesis of diseases caused by human coronaviruses have been studied using as a model the murine coronavirus, which causes hepatitis in mice (17). Nevertheless, coronaviruses have been overshadowed by other viral infections since they were not associated with human diseases, except for the common cold.

Before the epidemic of SARS-CoV, the coronaviruses were of significant interest in veterinary medicine because they infected mammals and birds, leading to respiratory and sometimes neurological diseases (3). When in the spring of 2003, it became evident that a new human coronavirus caused severe acute respiratory syndrome (SARS); coronaviruses acquired a new status of interest in human medicine and are considered “emerging pathogens” (4). The transition of the SARS coronavirus (SARS-CoV) from animals to humans underlines intriguing inquiries about coronavirus evolution and species sensitivity.

Despite the very sophisticated technologies implemented in producing new safe and effective vaccines (5, 6), hygiene has a complementary but essential position in confronting the pandemic.

Because of the nature of the virus, the transmission is either direct through respiratory droplets or indirect via contaminated surfaces which indicates the important of personal hygiene. Proper hygiene practices, including covering cough and sneezes, regular hand washing or showering before swimming act as an additional barrier preventing the spread of various diseases (18–20). Hygienic practices suggested from WHO as precaution measures for the effective control of the disease and against its spread apart from personal hygiene include the use of 70% ethanol as a disinfectant on hands or surfaces, use of personal protective equipment like medical masks as well as self-isolation or quarantine, general lockdowns and social distancing (21–23).

This pandemic has also challenged healthcare workers that ought to comply with new guidelines and hygiene standards in order to avoid occupational risks (24), before and after the development and administration of vaccines. The development and administration of vaccines might have not been effective against onward transmission of every variant once infected, but managed to prevent serious infections and thus, lowering the number of hospitalization (25).

Through this scoping review, it is worth investigating the scientific literature on SARS-CoV-2 and COVID-19 regarding hygiene and hygienic measures. Most research papers and reviews focus on clinical or therapeutic issues, epidemiology and other related topics. While during the pandemic surveillance systems to identify SARS-CoV-2 outbreak trends and prevalence included the detection of virus load in untreated waters (26), few studies only describe enough hygienic measures such as sufficient surface disinfection, hand washing or indoor air quality, and even less about the pathogen spreading by the fecal-oral route due to the cross-contamination of foods. The question about fecal-oral transmission of COVID-19 was raised from Bartram et al. and Bonanno Ferraro et al., since virus load is detected in sewage waters such as in WATER5 category of water related diseases and their modes of transmission (26, 27) and showcase the importance of sanitation and basic hygiene practices.

Human coronaviruses, such as SARS-CoV is known to be shed in the feces of infected individuals and remain viable, facilitating fecal-oral transmission (7). Nevertheless, in the case of SARS-CoV-2 infection there is only little information on the possibility of the virus to spread via fecal-oral but also via aerosols-droplet routes (8, 9). Studies reported the presence of viral RNA in feces of patients (10–13). The reasoning behind its transmission state that the viral RNA has been found in multiple body fluids, sometimes for longs periods of time. Yet, angiotensin-converting enzyme 2 (ACE2) cell receptor has been found to be expressed in various tissues of the human body, and reinforce the viral infection at these sites (28).

Recently, the next generation sequencing (NGS) of SARS-CoV-2 from stool of patients by analyzing mutational variations confirm the presence of SARS-CoV-2 in stools, its mutational shifts and thus, its role in fecal-oral transmission (14). SARS-CoV-2 RNA was found in feces starting on day 5 of illness and peaking on day 11. It is worth noting that only a few people remain positive for viral RNA even after 30 days of illness (14). This long duration of detection of SARS-CoV-2 RNA in feces compared to respiratory samples may ultimately support the fact that SARS-CoV-2 is actively replicating in the patient's gut and therefore fecal–oral transmission may occur after viral clearance in the respiratory system (15). In a recent study, amplification of the full genome/nearly full genome and identification of the complete virion morphologically using TEM analysis indicated the fecal excretion of the virion (16). However, the viability of the SARS-CoV-2 virion in feces needs to be confirmed using extended cell culture and animal studies. In this vein, it is also important to consider the viral load in relation to its presence in the intestine, as a higher viral load may be involved in the process (17, 29).

The authors aspire to provide an overview that maps the information covering these aspects of the subject related to the hygienic point of view in synthesis. This is -by definition- what scoping reviews do (30, 31). To this end, the present review tries not to exhaust with such issues as the use of masks or hand washing but rather to stress the impact of these practices on various environments, including food production facilities, retail shops and restaurants. The discussion about the virus's variants, the transmission routes, the disease's pathophysiology, and, of course, all the proper hygienic measures is endless. Our point of view is more practical and focuses mainly on applying all these to specific environments.

## 2. Materials and methods

### 2.1. Search strategy and eligibility criteria

One scientific database was used to identify articles relevant to our topic to extract research papers, review articles and manuscripts refer to the following keywords/phrases/nouns separately or in various combinations: SARS-CoV-2, hand washing, oral-fecal route, food, droplets size spanning from January 2019 to August 2021. The database used was PubMed, which includes over 35 million citations for biomedical literature from MEDLINE and life science journals. According to Gusenbauer and Haddaway (32), the PubMed database can be used for a systematic synthesis to conduct review research or meta-analyses and subsequently characterized as principal search systems. Moreover, PubMed's advantages as an academic search system are search settings, repeatability, advanced search field, multidisciplinary subjects, open access results, language options, AND/OR functionality (32, 33).

The methodology for this scoping review uses particular keywords for the search in the international academic database PubMed. The following search terms were used in the database' Advanced Search' feature, using the Title-Abs-Key search query: “hygiene and hygienic measures against SARS-CoV-2.”

The search was limited to English peer-reviewed manuscripts with the criteria mentioned above. Articles were excluded if we had not accessed them and were associated with clinical studies addressing the clinical picture, hospitalization and treatment, epidemiological studies from various countries concerning data such as the total number of cases and mortality rates, studies of molecular biology concerning the genetics and the virulence of the virus, masks and irrelevant to hygiene papers which were beyond this review's scope.

### 2.2. Data extraction and analysis

**Figure 2:**
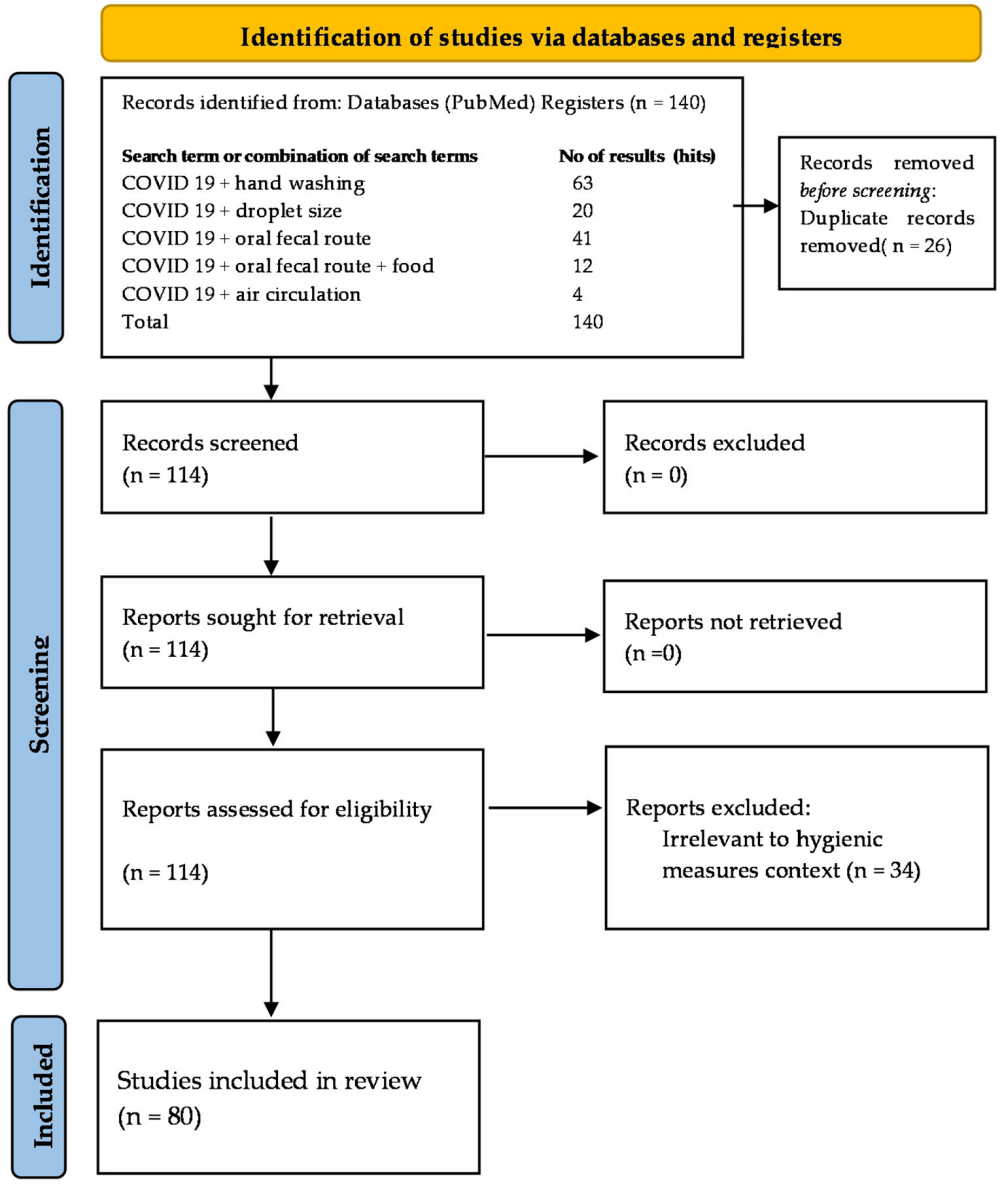
Flowchart of the literature identified, screened and included in this review according to the PRISMA guidelines (Number of hits on PubMed on the August 30, 2021) (34, 35).

All articles were extracted into bibliographic citation management softwares, EndNote library and Mendeley, duplicates were discarded, and exclusion criteria were applied by screening titles, abstracts, and full papers. A total of 140 review papers were selected. From these, 26 were discarded as duplicates and another 34 as irrelevant, leaving 80 review papers for data extraction. To establish a baseline for further analysis, the following section of the review discusses the structural and functional properties of the virus, as well as the epidemiological factors impacting its modes of transmission. Some -very few- data and comments about mask-wearing were unavoidable, although mass-wearing as a topic is not included in the scope of this review. The search result was summarized in the PRISMA- Preferred Reporting Items for Systematic Reviews and Meta-Analyses extension for Scoping Reviews (PRISMA-ScR) flow diagram (Figure 2) and checklist (Supplementary material) (35).

## 3. Results and discussion

### 3.1. General aspects

#### 3.1.1. Structure and properties of SARS-CoV-2 virus

Because of the presence of spikes on their surface, the coronaviruses have a characteristic crown-like shape observed in the electron microscope, to which they owe their name “corona,” meaning crown in Latin and the name was given in 1968 (36, 37). Coronaviruses are significantly enveloped, single-strand, positive sense RNA viruses ranging from 60 nm to 140 nm in diameter, with a genome about 27–32 kilobase size, with spike-like projections on its surface (34). According to genetic sequence, the Orthocoronavirinae subfamily has been divided into four genera (subgroups), the alpha, beta, gamma, and delta coronaviruses. The seven human CoVs (HCoV) which are known so far, belong in two of these genera: alpha coronaviruses (HCoV-229E, and HCoV-NL63), and beta coronaviruses (HCoV-HKU1, HCoV-OC43, MERS-CoV, SARS-CoV, and SARS-CoV-2) (38, 39).

Four of the capable infection of human coronaviruses (HKU1, NL63, 229E, and OC43) cause mild forms of respiratory disease in immune-competent patients. The 9, 10 SARS-CoV2, SARS-CoV, and the Middle East respiratory syndrome coronavirus (MERS-CoV) are zoonotic and infect humans, causing severe respiratory infections only by transmission from animals. The SARS epidemic in 2002 and 2003, caused by SARSCoV, showed a 10% fatality rate, while the MERS epidemic, caused by MERS-CoV in 2012, showed a 4% fatality rate. The estimations of the COVID-19 fatality rate vary significantly. These estimations were derived from surveillance data, calculated by crude models, and varied from <0.1% to over 25%, depending on the country and metho. Due to mutations and recombination of their genetic material, coronaviruses can adapt to new environments altering, thus, in a very efficient way, their host specificity and their tissue tropism (40–42).

Typically, the coronaviral genome contains genes coding for four structural proteins, namely, the spike (S), membrane (M), envelope (E), and nucleocapsid (N) proteins (43–47). Research findings strongly suggest that the S protein plays a crucial role in overcoming interspecies barriers, hence achieving interspecies transmission from animals to humans (39, 45).

When on January 10, 2020, the SARS-CoV-2 genomic sequence was detected. It appeared as a new form of beta-CoV (48). The genetic identity between the sequenced samples obtained from the outbreak's origin in Wuhan matched by more than 99.98% (49). Genetically, SARS-CoV-2 was reported to be more similar to SARS-CoV (50). It was determined that human ACE2 is a receptor for SARS-CoV-2 and SARS-CoV (51), with the significant difference that the SARS-CoV-2's S protein bond to human ACE2 is weaker than that of SARS-CoV, solidifying the theory that SARS-CoV-2 induces milder disease manifestations in patients than that does SARS-CoV (52).

ACE-2 receptors are expressed in many tissues; however, most are present in the alveolar epithelial type II cells (53). In addition, gene ontology enrichment analysis showed that the ACE-2-expressing epithelial cells have high levels of multiple viral process-related genes, including regulatory genes for viral processes, life cycle, assembly, and genome replication (54, 55). All these features strongly support the hypothesis that the ACE-2 receptor mediates SARS-CoV-2 replication in the lung (55). SARS-CoV-2, through binding to the ACE-2 receptor, down-regulates the ACE-2 intracellular signaling (mitochondrial assembly receptor), causing inflammation, vasoconstriction, and fibrosis in the lung (56).

### 3.2. Modes of transmission

With no scientific reports about the ≪moment≫ or the patient zero of SARS-CoV-2 infection, considering that (i) the human SARS-CoV-2 and bats' coronavirus express about 96.2% genomic similarity and (ii) the location in which a plethora of human SARS-CoV-2 infections was confirmed, for the first time, was a wet market in Wuhan, Hubei Province (China), a plausible theory of animal-to-person transmission was formulated (40). It was speculated that human SARS-CoV-2 might have been transmitted to humans from bats through other mammalian hosts (53). Besides, many studies have implied or proposed that snakes (57), turtles (58), pigs, ferrets, cats, pangolins and non-human primates could be possible intermediate hosts for SARS-CoV-2, with some of these theories being disproved by other researchers (59). Nevertheless, the growing number of infected humans in the community, who had never visited a particular wet market, indicated the shift of the transmission mode to a direct person-to-person spread.

**Figure 3:**
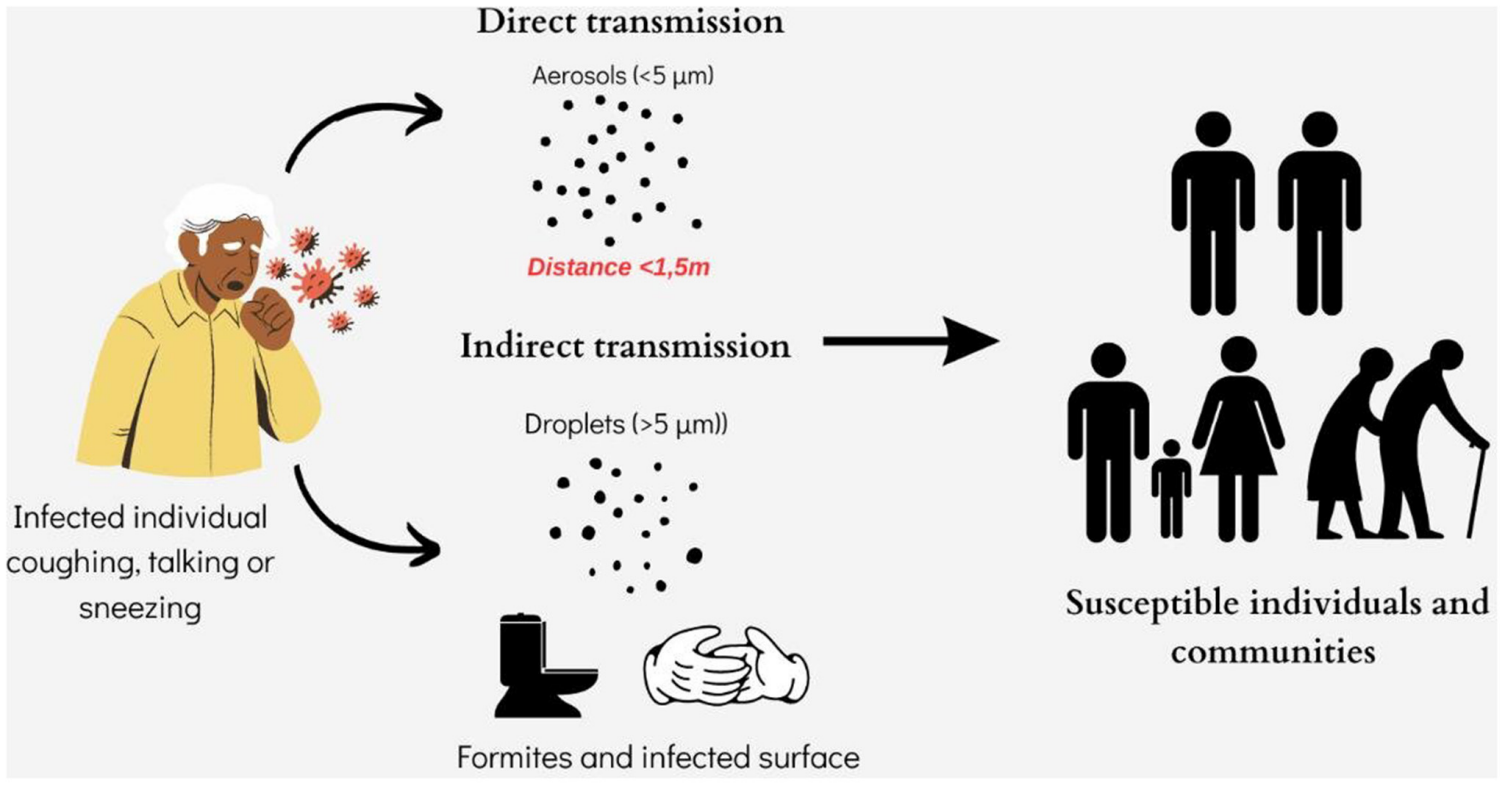
SARS-CoV-2 modes of direct and indirect transmission.

Currently, the 2019-nCoV spreads between individuals primarily through saliva droplets or discharge from the nose (respiratory droplets produced by an infected person while sneezing, coughing or talking and staying a short distance from another person (Figure 3). Depending on oral hygiene, each person's saliva is a bio-mixture, which physiologically contains crevicular fluid, desquamated oral epithelial cells, and microorganisms (60). Also, in pathological occasions, which vary from moderate to severe, these discharges may contain respiratory secretions, gastric acid from reflux, food debris and blood (60, 61). Previous studies showed that saliva has a high concordance rate of > 90% with nasopharyngeal specimens in detecting respiratory viruses, including coronaviruses (62, 63). Given the fact that the official pathogen detection (case) of 2019-nCoV infection is the confirmation of virus nucleic acid from throat swabs (64) and that the oral cavity is an entrance and an exit of the body and also that it is anatomically a common element of the respiratory and digestive tracts, is not only a plausible but also a logical view, that many researchers consider that saliva and nasal secretions (as recurrent pharyngeal-throat secretions) are the vehicles of the virus to spread the infections from human to human (65, 66).

It follows that the main path of transmission of SARS-CoV-2 is the “instantaneously - direct contact” by (a) infectious respiratory droplets penetration at one or more “gates” of the personal visceral skull, such as the mouth, nose or eyes and (b) by direct contact with fomites with SARS-CoV-2 in the immediate environment around the infected person (even rubbing or shaking hands) or surfaces contaminated indirectly with infectious respiratory droplets and afterwards next contact with own nasal cavity, oral cavity or eyes (67). Scientists published studies confirming that prolonged close contact is the leading risk factor for transmission and that the risk of infection is much higher in contacts within indoor spaces compared to outdoor contacts (67, 68).

The virion consists of a in its extracellular phase, as opposed to its intracellular structures associated with viral replication (69). Elimination of infectious non-retroviral RNA viruses after recovery is known to lead to the development of immunity, however without concomitant elimination of viral RNA (69–71). Persistence of viral RNA has been accepted, particularly at sites associated with the immune system, but may also affect other sites including lymphoid tissue and the gut-associated lymphoid tissue (GALT) system sits in the intestinal wall furnished by immunological elements and consists of one of largest immune organs in our body with well-organized lymphoid tissues, such as mesenteric lymph nodes and Peyer's patches, as well as lymphocytes in the intestinal epithelium (72, 73).

However, infectious virions are not always recovered in these secondary sites and the viral RNA is prone to degradation (74). Nevertheless, recent studies report the long-term persistence of non-retroviral RNA able of producing replication in the cytoplasm when the immune capacity is relaxed (75). However, the question arises about the role of persistence of viral RNA, often without evidence of infectious virions. In this vein, the hypothesis of reverse transcription by cellular enzymes has been postulated as a mechanism of persistence for the non-retroviral RNA viruses as endoviral components in the cytoplasm. Yet, capsid RNA capping may protect the RNA of negative-strand viruses, while attachment to other membrane components may contribute to the protection of positive-strand RNA viruses (76, 77). The development of more accurate techniques for virus detection will certainly contribute to gaining further knowledge about the persistence of RNA viruses, as the lack of detection of infectious virus could be related to the sensitivity of the assays applied (78). It is reported that innate immune mechanisms can affect intracellular viral replication and induce viral RNA degradation, but the adaptive immune response of virus-specific antibody and T cells is known to induce complete clearance of infected cells. However, it is noteworthy that the survival of infected cells is often associated with forms of viral mutations in genes encoding proteins required for assembly or replication that support persistence by evading the adaptive immune response (77–79).

Tracing studies demonstrated that SARS-CoV-2's viral RNA has not only been found in upper respiratory tract secretions but in many other body fluids and excretions such as feces, blood and (rarely) urine (38, 80, 81). Furthermore, considering the following facts: (a) angiotensin-converting enzyme 2 (ACE2) is the cellular entry receptor of SARS-CoV-2, (b) ACE2 is found in the absorptive enterocytes of the ileum and colon (and is prone to be infected by coronavirus), (c) studies found that ACE2 was highly expressed in the small intestine, especially in proximal and distal enterocytes and therefore, the digestive system can be invaded by SARS-CoV-2 and serve as a route of infection, raises the issues that oral-fecal transmission of the virus or inadvertent human-to-human transmission via the fecal route are presumably also possible (Figure 3) (82–84). Especially when some studies show that SARS-CoV-2 RNA seems to be present later and persists longer in fecal samples than in samples from the upper respiratory tract (15).

Although limited in number, certain studies report that it recovered SARS-CoV-2 from stool samples from patients or that SARS-CoV-2 invades and infects gastric, duodenal, and rectal glandular epithelial cells (10, 84, 85). These previous studies demonstrated that in more than 20% of patients with SARS-CoV-2, viral RNA had been detected in feces, even after test results for viral RNA in the respiratory tract converted to negative. This observation raises the hypothesis that a potential fecal-oral transmission is plausible and can last even after viral clearance of the respiratory tract and if this is ever epidemiologically documented then a strong recommendation should be installed to prevent further transmission via the oral-fecal route if rRT-PCR result of a fecal sample remains positive (15).

Unlike abovementioned results, another study shows that the virus (as proven by viral culture), despite the recovery of high viral load from the throat- and lung-derived samples, was not present in fecal samples (were negative to PCR results) (80). No study so far has either proved or documented fecal-oral transmission. Neither any study has been able to correlate and prove that the presence of viral RNA in fecal samples of COVID-19 patients or asymptomatic individuals marks equal infectious potential (86).

Possible intrauterine transmission of the virus has been studied in cases of delivered a newborn infant via cesarean section from a COVID-19 þ (+) mother (87–89).

### 3.3. The global impact of hand hygiene

When writing this review, new reports are being published, shedding light on the COVID-19 disease or raising debates Hand washing offers some protection in the dispersion of the virus and this simple act or habit has received considerable attention since the pandemic's beginning. Since the advent of the COVID-19 pandemic, hand washing has emerged as one of the fundamental disease control strategies, which must be faithfully followed by the global population (90–92).

It must be stressed, however, that hand hygiene (HH) is a long-term challenge, and it is not something unheard of for humanity which has faced several pandemics in the past. Significant historical data confirms the origins of HH and the practical use of disinfectants (from Homer in 800 BC to Scheele's discovery of chlorine and Semmelweis's concept of HH) (93–99). This way, HH was established as a practice of vital importance. In the second half of the nineteenth century, Louis Pasteur's Theory of Germ infection was accepted, giving birth to infection control practices that began evidence-based practice (100).

In the wake of the growing burden of healthcare-associated infections, the World Health Organization (WHO), in its 2009 international campaign “SAVE LIVES: Clean your hands,” put HH at the heart of infection prevention and control in healthcare settings. This is because it is “the simplest and most effective measure of prevention” (91, 101). HH refers to removing the fauna from the epidermis of the hands, either by washing them with soap and water or by disinfecting them with an alcohol-based antiseptic. Its goals are to discontinue the cross-infection of patients with microorganisms, prevent infection of both patients and healthcare professionals and prevent the colonization of the epidermis of the hands with potential pathogens. To achieve these goals, it is necessary to comply with the instructions for correct HH wherever indicated fully. The WHO has put forward “the five steps to Hand Hygiene” approach for an apparent and easy-to-remember reference of the indications where HH is necessary. This defines a clear frame of time and place where HH is necessary but also where it is not, and so facilitates the training of healthcare professionals and minimizes personal interpretation as to the need for HH. According to the 5-step approach, HH must take place regardless of the use of gloves: ≪before contact with the patient≫, ≪before any aseptic procedure≫, ≪after the exposure of a patient to biological liquids≫, ≪after contact with the patient≫ and finally, ≪after contact with the lifeless environment of the patient (external body surfaces)≫ (102). Compliance with the above guidelines is not a personal choice or common sense but of strict professional obligation in healthcare environments.

Correct observation of the rules of HH was and is the most effective strategy for preventing microbial transmission in healthcare environments and preventing infections related to healthcare provision (16).

HH is also defined as removing microorganisms from hand surfaces that can cause infection through transmission among people, be they patients or healthy individuals. Using gloves does not negate the need for HH, which must be practiced before and after their use (103).

It is therefore deemed necessary first to discuss the essential structural elements of the COVID-19 before shedding light on the role of HH as a first line of defense against its spread. Numerous studies have described the structure of SARS-CoV-2. It is an enveloped positive-sense single-stranded RNA virus (+ssRNA) assembled by three building blocks: the RNA, the lipid bilayer envelope and the membrane proteins forming the nucleocapsid. The lipid bilayer, consisting of cholesterols and phospholipids, protects the virus and assists its spread and cellular invasion. The self-assembly process of the virus involves non-covalent “like Velcro” interactions between the RNA, the proteins and the lipids, making the viral particles' disassembly harsh (81, 91, 100–108).

If, based on its small size (50–200 nm), we accept that the SARS-Co−2 virus is a nanoparticle, then by default, we accept its capacity to create multiple complex interactions with various surfaces, depending on the materials with which it connects. That is why the virus interacts differently with skin, steel, wood, cloth, paint or porcelain. The structure of the outer layer of each material plays a significant role in the potential attachment of the viral particles. The smoother the surface, the less adhesive it is for the virus (steel, porcelain and some plastics such as Teflon), while rougher surfaces (wood, cloth, and skin) interconnect quite easily with viral particles (93). The skin is an ideal surface for a virus due to its organic composition. Its cells' protein and fatty acid content interact with the virus by forming hydrogen bonds and through hydrophilic interactions (93, 105).

An infection in that case begins with a person carrying the viral particle on hands, touch ones face. As a result, the virus may invade the mucus membranes of the oral cavity, the nose or the eyes causing the infection, unless the immune system destroys the particle. Therefore, handshaking, kissing and sneezing favor the spread of the virus between individuals. Since most humans touch their faces once every 2–5 min, it follows that hand washing is the only means of protection. Simply rinsing the virus off the skin with plain water is adequate.

On the other hand, soap and water act entirely differently, as soap contains fatty substances known as amphiphiles, some of which are structurally remarkably similar to the lipids of the viral membrane. The soap particles “compete” with the lipids of the viral membrane in the same way that they remove everyday dirt from the skin (93, 94).

Alcohol-based products, which comprise most hand disinfectants, contain a high percentage of alcohol (usually 60–80% ethanol) and kill the virus similar to soap (95, 96). However, soap is better because only a small quantity is needed to cover the whole surface of the hand through rubbing quickly. At the same time, one literally must submerge the virus in alcohol for a brief moment in order to kill it. So rubbing alcohol wipes and solutions onto your hands does not guarantee adequate coverage of your hands' skin with ethanol in all areas (97).

**Figure 4:**
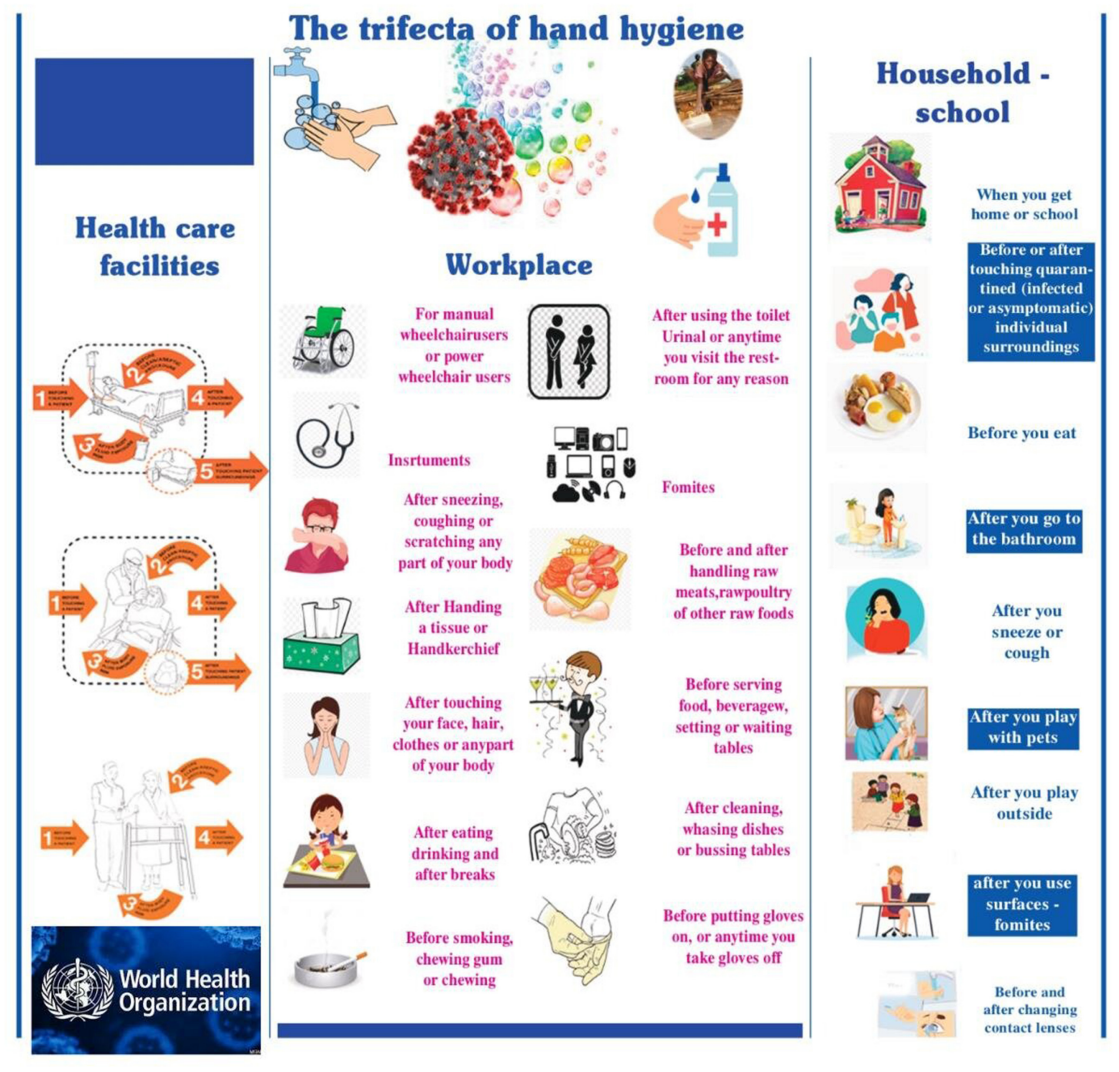
The trifecta of hand hygiene: health care facilities (not only hospitals, but also where individuals with mild symptoms are hosted), workplace (referring to asymptomatic carriers) and community (geographical and cultural locations of humans), such as schools or households.

In Figure 4, an effort is made to document the possible ways human hands are infected and the key places where hand washing, or disinfection is crucial. According to this, humans are placed in the trifecta: healthcare facilities (not only hospitals but also where individuals with mild symptoms are hosted), workplaces (referring to asymptomatic carriers) and communities (geographical and cultural locations of humans), such as schools or households. In all the above cases, human hands act as a conduit for the intrapersonal and interpersonal transfer of viral loads of the SARS CoV-2 affecting it considerable importance between the environment and the body rendering the dynamics of hand hygiene and the factors affecting it considerable importance.

Due to the absence of pathogen-specific therapeutic medicines, the current SARS-CoV-2 control model is “prevention is all we have.” The US Centers for Disease Control and the World Health Organization put frequent hand washing at the top of their SARS-CoV-2 prevention guidelines for the public (98, 99).

A great example highlighting the impact of hand hygiene is the vigilance of Koreans during the severe Middle East Respiratory Syndrome (MERS) outbreak in May 2015, which occurred in several regions of South Korea (109). During the outbreak, 186 confirmed cases were reported throughout the country, according to the KCDC (109). There is no effective vaccine against this global threat, whose fatality rate is ~35% (109). Correct hand washing is considered the first and most vital way to prevent the spread of MERS (110). In Korea, as of July 4, 2015, no more confirmed cases were reported (111). This means that the MERS outbreak may have increased Koreans' awareness of the significance of hand hygiene, and changed hand-washing behavior among healthcare workers (112), elementary school to high school pupils (113) and adults (114). The behavioral response was high in the case of SARS-CoV-2 in Korea. Citizens reported practicing safety measures, such as faithfully observing facial masks when leaving home and practicing hand hygiene. These measures aim to minimize the virus's spread, which helps protect the healthy population against viral infection from SARS-CoV-2 (115).

One would therefore expect that today, in an age of evidence-based medicine, with the abundance of information available and the strong guidelines issued regarding the absolute need for hand hygiene, 100% compliance could be taken for granted, not only in the health care facilities but also in all areas described in Figure 4. Indeed, many studies and bibliographical reviews show that the COVID-19 pandemic has reinforced the importance of this scoping review comprehensively addresses and provides valuable information on the crucial topic of hygiene standards and measures to combat the COVID-19 pandemic. Moreover, there is a compelling call for international and national public health bodies to delve into the relationship between outdoor and indoor air quality and its nexus with COVID-19's trajectory. It is expected that environmental factors and pandemic dynamics may be critically connected.

In addition, the review underscores the significance of delving into the interplay between COVID-19 and the food industry. Investigating food packaging materials and the virus's survival in food commodities could yield profound insights into global transmission patterns. Such research stands to enrich our understanding of viral dissemination across geographical borders.

In conclusion, this scoping review enriches our comprehension of hygiene measures in countering the pandemic. It underscores the necessity for interdisciplinary collaborations and targeted research to unravel the multifaceted dimensions of COVID-19's impact on various fronts.

Washing in hospitals and dramatically improved hand hygiene performance rates, especially in the area of hospitals or health care settings in general (95, 116–119). However, as to the question of whether this public health emergency has triggered a behavioral change in each member of the public so that the observation of simple HH rules becomes an instinctive/reflexive action (in an ideal neurological world, there would be an activation of the parasympathetic autonomous neural system), with no peripheral factors affecting compliance, the answer is a resounding no. Unfortunately, compliance with proper hand hygiene, even in hospitals (including visitors and health care workers), could be better (120–124). Population-based studies on hand hygiene, considering the COVID-19 pandemic, indicated that hand-washing behaviors are not satisfactory (116–119, 124–133).

When trying to ascertain the reasons and factors affecting noncompliance to HH in health care settings, it is helpful to note the December 2020 announcement by WHO that: ≪One in four hospitals around the world do not have running water and basic hygiene and disinfection services, thus exposing healthcare professionals and patients to increased risk of infection from COVID≫ (134). According to a joint WHO and UNICEF report based on data from 165 countries, ~1, 8 billion people visit or work in hospitals without running water or waste disposal systems. ≪Working in a hospital without running water, waste disposal system and disinfection amount to sending healthcare professionals to work without individual protective equipment≫, stressed Director-General of the World Health Organization Tedros Adhanom Ghebreyesus. He emphasized that running water, waste disposal systems and disinfection are vital in the fight against COVID-19 but that there are still many problems to overcome in less developed countries (128).

Executive Director of the United Nations Children's Fund Henrietta H. Fore stressed, ≪By sending healthcare professionals and patients into facilities without clean water, safe toilets or even soap, we are risking their lives≫. ≪This was already true before the COVID-19 pandemic, but this year's pandemic has made it impossible to ignore the problem≫. While still in shock regarding the above data and to record the complex reasons for poor compliance with hand hygiene among nurses and other healthcare workers, an overview of the literature suggests that the following are part of the picture (Table 1) (129–131).

**Table 1:** Reasons for poor compliance with hand hygiene among nurses and other healthcare workers (119).

**List of reasons**
Lack of awareness in healthcare professionals regarding the importance of HH in preventing infection
Workload
Understaffing
Lack of leadership
Difficulty accessing hand hygiene resources such as running water and soap
Skin irritation due to frequent hand washing without moisturizing care
Lack of necessary antiseptic solutions
Indifference and negligence
The fallacy of glove protection
Urgent patient needs coming first
Cultural background and religious beliefs

Identifying the reasons behind noncompliance to HH on an individual level is difficult. Human behavior is highly complex and is affected by multiple interrelated influences ranging from biology and the environment to education and culture. So, it is best to consider individual behavior and the obstacles to putting theory into effective practice. Ensuring a positive attitude toward HH and improving awareness through attending educational programs and seminars will enhance knowledge regarding HH and increase its application in everyday life. Also, interventions to improve hand hygiene do not have to be extraordinarily intensive. Easy access to water and soap or hand-hygiene products can improve hand hygiene compliance.

In healthcare facilities, HH training would become even more effective if specific groups of healthcare professionals and other healthcare workers (cleaners, private nurses) were targeted to ensure that all individuals involved with the patient receive appropriate instruction. Applying the guidelines by as many groups of workers as possible will become a healthy example to follow, not only for other employees but for the patient's immediate environment as well.

According to the adage, the Baconian principle “Knowledge is power” dictates the need for all involved participants (management, infectious disease group, secondary staff/satellites, employees of all specialities, visitors to health care facilities) to embark on a joint effort to improve awareness. Putting theory into practice is of critical importance. Through repeated, varied, and compulsory training programs, employees should be regularly reminded of the proper procedures to avoid non-compliance in the stressful and exhausting work environment. This way, through monitoring and rewarding employees, correct HH will be practiced at the highest possible level, thus preventing cross-contamination and the spread of infection in healthcare settings and avoiding their subsequent consequences.

Before planning public health and hygiene strategies, the authorities or the management must be aware of the characteristics and particularities of each target group. This means that interaction must be initiated with the group/population to which the health and hygiene guidelines are targeted in a supportive and non-judgmental way so that the group would design the whole strategy to improve compliance.

Research has proven that hand washing frees individuals psychologically (116, 132). We must instill beliefs of equivalent magnitude in the behavioral attitudes of people regarding physical health. Hand hygiene is inextricably linked with infection prevention.

“If a pandemic did not change hand hygiene, no amount of vigilance ever will. Awareness of hand hygiene was at an all-time high as providers were afraid for their lives, but hand hygiene still decreased. We must look at it not as a people problem, but as a system and human factors issue,” said Adam Webb, MD, Chief Quality Officer, Emory University Hospital (117).

### 3.4. The importance of air hygiene

So far, there is limited data on the aerodynamic properties of the SARS-CoV-2 virus concerning the different transmission routes through aerosols and droplets. CDC and WHO accept that respiratory excretions should be classified into small and large droplets, a theory first proposed by Wells (67, 118, 119, 133, 135). Most researchers agree that 5–10 μm is the upper cut-off limit of the size of the small droplets (also described as aerosol) and that they can hover for more extended periods. Thus, they can cover more considerable distances (136–141). Larger droplets (size > 20 μm) are heavier and have shorter trajectories, covering shorter distances from their source (usually 1–2m). Droplets with 10–20 μm size represent an intermediate class, and they can either remain hovering or land on various surfaces, depending on the conditions. Sometimes aerosol droplets can shrink to form the so-called “droplet nuclei” (136–143). The fate of the particles is determined not only by their size but also by their speed, density and composition, momentum, the humidity of the environment and micro-currents of atmospheric air (118). The formation and aerodynamics of each droplet category are crucial factors for the transmission of SARS-CoV-2 and have two significant consequences. If the virus is transmitted mainly by the larger droplets, then the masks, the face shields and the social distancing should be more than enough to avoid the infection. If aerosols transmit the virus, then the masks, the face shields and the social distancing cannot protect from viral particles that can hover for long periods and are carried by air currents. The larger droplets are heavier, and gravity shortens their trajectory while aerosol carrying the virus can easily reach the bronchi. The intermediate-size droplets have some chance to pass the glottis barrier (139, 142, 143).

Most of these studies on droplets and aerosols do not take into consideration the survival rate of the virus, which decreases proportionally to the distance of the infected person (source), but also viral particles are destroyed by the solar radiation, the drying of the droplets, the temperature and by virucidal substances like ozone which generally exist in the air (144–146). These factors significantly reduce the concentration of the virus in the atmosphere and the possibility of infection. Environmental factors do not affect all respiratory viruses to the same extent. Furthermore, the exact location of the inflammation of the respiratory tract and the clinical disease affects the size of the droplets, their emission rate and composition, consequently determining the viral survival and spreading. Laboratory experiments under controlled environmental conditions and field monitoring have revealed valuable data for estimating airborne transmission of respiratory viruses. For example, SARS-CoV is considered higher than Influenza (147). On the other hand, although speaking and coughing produce aerosols and viral RNA is isolated from air, these facts are not enough to establish transmission because infection also depends on other factors such as the route of exposure, the size of the droplets, the period of exposure and course the immune response of the host (118, 148–150).

The dose-response relation of SARS-CoV-2 infection remains unclear. Researchers claim that the virus can be transmitted in closed spaces with poor air circulation, low humidity and high temperature in due course (151–155). However, the opinion that aerosol can transmit the virus is debatable among different research groups (156–162). WHO accepts that transmission by aerosol is possible only in healthcare facilities due to medical interventions such as intubation, mechanical respiratory support and administration of medicines by nebulizer (158). The aerosol transmission mode has been thoroughly investigated in hospitals and the community ever since (159).

Several hospital studies have researched and monitored the presence of SARS-CoV-2 in samples of air taken from hospital wards for COVID-19 patients (160–162). In a study of the aerodynamic characteristics of COVID-19 by Guo et al. (163), the air and surfaces of intensive care units (ICU), as well as other areas hosting COVID-19 patients, were studied. In addition, three different sampling points were chosen: (a) the patient hospitalization area, (b) the doctor's offices and (c) areas neighboring the exit points of air. The SARS-CoV-2 genome was identified in samples taken from all three points with a development rate (of 8/18, 44.4%) in the patient hospitalization area and (5/14, 35.7%) in areas near the exit points of air.

Most published studies explore the hypothesis that SARS-CoV-2 transmission may be airborne (through aerosol), but there is no tangible evidence. The methods of air sampling, the exceedingly small number of samples, the methods of genetic identification of the SARS-CoV-2 genome and the protocols for the estimation of possible airborne transmission have yet to be evaluated and verified.

In addition, there have been few studies regarding the transmission of viral loads by asymptomatic individuals (163–165). These individuals are unaware they are carrying COVID-19 since they do not have symptoms of a respiratory viral infection, such as coughing or sneezing. The expulsion of droplets, in their case, is made during everyday interactions like speaking, laughing and singing. Published data worth mentioning (166, 167) shows the possibility of airborne transmission through aerosol containing an active SARS-CoV-2 viral load. In this study, people participating in a choral practice in Mount Vernon, Washington, who had stated they had no symptoms of a respiratory viral infection, observed social distancing and limited their physical contact with each other, practiced for 2 h. After the practice, 45 of the 60 members were found positive for SARS-CoV-2 or had COVID-19 symptoms. Three were hospitalized, and two died.

Also, worth mentioning in this review is a study researching the production of air droplets by a group of healthy volunteers during coughing and speech (167). In this study, the distribution of the droplet size, the speed and distance of their transmission and the airborne time concerning the level of air ventilation were measured. It was found that small droplets (1–10 μm) prevailed during coughing, while during a speech, they were the only ones isolated and recorded. As for their speed and trajectory, it was no surprise that at the beginning of coughing, the large droplets (typically 500 μm in diameter) descended immediately toward the ground within 1 s due to gravity. The small droplets with a typical diameter of 5 μm took 9 min to settle on surfaces or reach the ground.

Furthermore, the production of nasal cavity droplets was studied during normal breathing and sneezing. In the former, there were no droplets from the nose, while in the latter, large drops originating mainly from the secretions of the nose and mouth dominated. Finally, the same study looked at the trajectory and movement of small droplets produced by coughing, through the air, using a simulator (spray nozzle from Medspray; Enschede, The Netherlands) to disperse a controlled quantity of small droplets into the air, reproducing the effect of coughing) in three spaces with different levels of ventilation: (a) no ventilation, (b) only mechanical ventilation and (c) mechanical ventilation supported by an open entrance door and small window. In the last case with the best ventilation, after 30 s, the number of droplets had decreased by half, while in the first case with no ventilation, this took ~5 min. The air drag calculation diagram shows that small droplets (5 μm) take about 9 min to reach the ground in the case of a light cough or calm speech (from an expulsion height of 1, 60 m from the ground). Droplets had decreased by half in a poorly ventilated room in 1.4 min.

The above study indicates the need for hygiene and good ventilation in indoor spaces to reduce droplets' time to stay airborne significantly. These findings are noteworthy since these poorly ventilated populated spaces, such as means of public transport and older people's homes, are the habitat of viruses. Of course, in such spaces, their transmission is relatively easy, regardless of precautions such as social distancing. The lingering respiratory droplets in such poorly ventilated spaces could contribute to the spread of SARS-CoV-2.

As such, due to the possibility that SARS-CoV-2 spreads essentially through aerosol-type droplets, research groups ardently advocate the benefits of an efficient ventilation system, possibly assisted by particle filtration and disinfection of the air. They believe such systems are necessary to drastically reduce the risk of infection in indoor spaces (144, 168).

In order to maximize the protection of the population against airborne transmission of SARS-CoV-2 or any other micro-droplet carrying airborne viruses, there are various requirements, as described below. They focus mainly on indoor spaces and government buildings since that is where the main transmission volume occurs (140, 169). In domiciles and apartments, the usual practices ensuring clean indoor air (e.g., isolating infected individuals, opening doors and windows and using portable air purification devices, where practical) must always remain in place.

**Figure 5:**
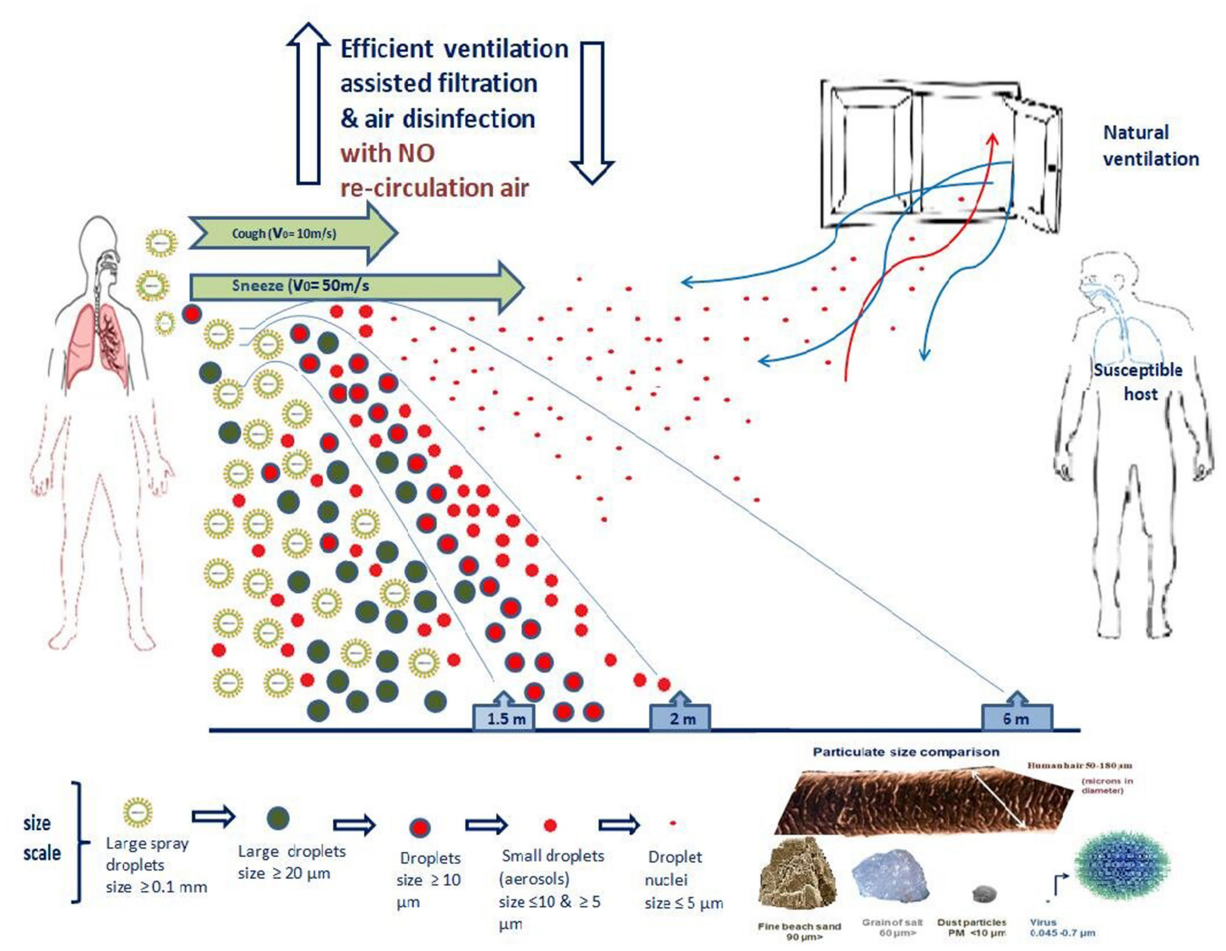
Aerodynamics characteristics of SARS-CoV-2 and “mechanical (level) control” of airborne infection.

The existing ventilation measures protecting against airborne infection can quickly be reinforced relatively cheaply to reduce infection and save lives. The options discussed below must always be applied in combination with other measures already in place (such as hand washing and the use of self-protection measures) for the reduction of infection via other significant mechanisms of infection since none of them can be excluded in any instance of exposure to the virus. The rest of this section will concentrate only on recommendations regarding “mechanical (level) control” as described in the conventional/traditional hierarchy of infection control (Figure 5) to decrease the danger of airborne infection. The HVAC system control strategies (heating, ventilating and air conditioning) can be modified to increase ventilation, to some extent, in high-risk areas at relatively low cost to diminish the danger of airborne transmission among passengers. However, this cannot be done simply with the ≪flick of a switch≫, as the HVAC systems are complex and usually designed for specific buildings with specific, standardized operation parameters. Besides the ventilation rate, many requirements must be considered, including temperature control and relative humidity, as well as the distribution and direction of airflow. These systems can be specially adapted as needed by HVAC technicians to diminish the danger of airborne transmission. Indeed, the ventilation guidelines of ASHRAE (The American Society of Heating, Refrigerating and Air-conditioning Engineers), REHVA, and SHASE (The Society of Heating, Air-Conditioning and Sanitary Engineers of Japan) have been updated to combat the spread of SARS-CoV-2 (142). Another example is modifying the ventilation system in a hospital ward to create a negative pressure isolation room (170).

Suppose there is natural ventilation through doors and windows or other openings. In that case, the potential rate of external airflow can be estimated using the CEN Standard, EN 16798-7: 2017 or other available protocols such as (171).

In naturally ventilated public buildings, other challenges will occur, especially in cold climates, but these can also be addressed to diminish the danger of airborne transmission. Additional heating may be necessary for some buildings to maintain heating comfort, especially if the individuals therein are vulnerable.

### 3.5. What about air re-circulation

Air re-circulation is a way to save energy. However, care must be taken, as it can carry air pollutants (including viruses) from one area connected to the same ventilation system to another, increasing the risk of airborne spread to spaces which would not otherwise have been contaminated. This concern had previously arisen in connection to the potential re-circulation of biological agents in cases of terrorist attacks, where the efficiency of eliminating air re-circulation was studied (e.g., by pumping 100% external air into the building) as a counter-measure for the release of toxic biological agents in indoor areas. Another study which created a model of the danger of airborne flu transmission in private cars also addressed the issue of eliminating air re-circulation in such cases (172, 173).

Particle filters and disinfection equipment in air re-circulation systems can reduce this danger. However, they must be deliberately designed to control the risk of airborne infection and be regularly serviced to maintain their effectiveness. Many systems have been designed with filters that remove larger particles which could adversely affect the mechanical operation of the equipment but are unable to withhold and remove microscopic particles (micro-particles/micron size), which are related to adverse effects on human health. Filter systems must be rated according to standards technology, which indicates their performance, and must be used to select appropriate filters (173, 174).

According to the above recommendations, during a pandemic, including the current COVID-19 outbreak, the air must not be re-circulated, as much as possible, to avoid the circulation of particles with a viral load throughout the indoor environment. If possible, this could be done by separating ventilated areas into multiple zones and operating the system with 100% outside air (OA). Re-circulation deactivation may be achieved by shutting the re-circulation vents and opening the outside ones. In systems where this is not possible, the quantity of outside air must be maximized and filtering radiation or UV microbicides must be applied to remove or eliminate any possible viral infection from the re-circulated air. In most hospital care sites, air re-circulation is not allowed at all, and it is mainly used in non-healthcare settings to achieve energy savings. At the de-centralized level (individual rooms), secondary ventilation/air circulation systems may be installed, and it must be ensured that they also provide fresh air ventilation (e.g., inductive units). If they exist, such systems must not be deactivated. However, other systems without the ability to bring in OA (e.g., split A/C units) should, if possible, be deactivated to prevent the potential spread of viruses between people due to airflow. When these systems are required for cooling, additional ventilation with fresh air must be developed regularly, and air cleaning/disinfection could also be beneficial.

Adding portable air cleaning/disinfection devices may be helpful in environments where improving ventilation is complex. In addition to hand hygiene and social distancing, the parallel reduction of airborne transmission using mechanical controls in hospitals and other public buildings will further protect health workers, patients, and the public.

### 3.6. SARS-CoV-2 in the food supply chain: the possibility and plausibility of orofecal transmission

The orofecal route of transmission describes the ways by which microorganisms from the digestive tract of one person enter the oral cavity of another person. This route can be direct through physical contact or indirectly through other materials such as vehicles. The packaging of the food or the food itself may harbor for longer or shorter periods of pathogenic microorganisms. In order to discuss the possibility and plausibility of the orofecal transmission of SARS-CoV-2, three conditions must be met: (i) the virus must be able to multiply or at least survive in the gastrointestinal tract, (ii) the virulence of the virus must not be affected by the processing of the food or the virus must be able to survive on the packaging of the food and (iii) compromised personal hygiene of food operators. Some other single-stranded RNA viruses, like noroviruses, are transmitted primarily through the orofecal route (175).

As previously mentioned, its viral particles, like SARS-CoV and MERS, use angio-tensin-converting enzyme 2 (ACE2) as entry to the cell. ACE2 is found in the absorptive enterocytes in the small intestine (mainly in the ileum) as well as in cells in the colon (44, 176, 177). Jefferson et al. (173) claim that ACE2 receptors are also found on cells in the gastric wall and the duodenum. These cells can host various viral species, such as coronavirus, rotavirus and norovirus. Hence SARS-CoV-2 can infect the human digestive tract, and diarrhea may follow as a clinical symptom. It is true that, for obvious reasons, emphasis has been given to the respiratory signs of the disease and the respiratory route of infection through droplets, and all precautions and prophylaxis (masks, social distancing etc.) focus on avoiding inhalation of infected droplets.

Wu et al. (15) report that in patients recovering from the disease, stool samples were positive in viral RNA for 11.2 (± 9.2) days after the respiratory samples were found negative (in total 27.9 ± 10.7 days after the first onset of symptoms). In their report, these researchers state that the presence of gastrointestinal symptoms was not associated with fecal viral RNA and insist that since the virus replicates in the intestinal tract for more extended periods than in the respiratory tract, the orofecal route of transmission by discharged patients who recovered from the disease cannot be ruled out, particularly in contained living premises like hostels, dormitories, trains, ships and buses. Parasa et al. (83), in a meta-analysis of 29 studies, found that the pooled prevalence of diarrhea among COVID-19 patients was 7.4%, much <20% observed in SARS patients. In their opinion, this difference could result from underreported gastrointestinal symptoms in the initial studies and concluded that the intestinal tropism of the SARS-CoV-2 is like the SARS infections. According to the authors, these findings make possible orofecal transmission and could explain part of nosocomial infections. In the same study, 40.5% of the patients who were favorable to the viral RNA respiratory samples also showed positive fecal samples. In general, the viral load of the feces was lower than that of the respiratory samples. Gupta et al. (176) report that after the clearance of the respiratory system in COVID-19 patients, the viral RNA was present in the feces from 1 to 33 days, with one patient's samples being positive for 47 days (178).

Although SARS-CoV-2 in the digestive tract has been proven, the exact mechanisms through which the virus overcomes the various hazards like proteases, low pH and bile salts remain unknown. According to a study, both S glycoproteins and the viral envelope must be resistant to these adversities due to heavy glycosylation (44). Other –yet unknown- processes taking place in the digestive tract may favor the infective action of the virus, as in the case of the bovine coronavirus, where the S glycoprotein must be cleaved by a protease so the virus can become pathogenic. However, in most studies, some part of the viral RNA is detected in the samples, and only a few studies report the detection of the whole virus in feces (38, 39, 179, 180). These findings provide a reasonable explanation of the expected rareness of occurrence (if any) of the orofecal transmission of SARS-CoV-2.

The most obvious way the infection of foods is by touching without gloves or prior hand sanitation, coughing, sneezing and talking without wearing a mask in a food-production facility. In these ways, either the food or its packaging will be infected. The virus can contaminate fresh products (vegetables, bakery products) or the packaging via an infected person. In such cases, the virus can be transmitted directly if it is transferred within a short time interval to the mucous membranes of the eyes, mouth and throat (180).

Orofecal transmission could lead to the same result. Since the virus can survive up to 4 h on a copper surface, up to 24 h on cardboard and up to 2 or 3 days on plastic or stainless steel (181), the packaging materials can act as vehicles in the orofecal transmission. Cooking and heat treatment kill the virus in the mass of the food, but refrigeration does not. Even if the virus is destroyed in cooked foods, the possibility of cross-contamination of other foods before cooking through direct contact or handling cannot be neglected. However, it must be noted that specific traditional food preparations and some eating habits (e.g., rawism) may not kill the virus or -even worse they may provide means for its survival. Scientific data on the survival of the virus in such conditions need to be included.

FAO/WHO and EFSA, in their 2020 documents, consider the possibility of transmission of SARS CoV-2 through food as “highly unlikely” (180). In principle, if all measures described in ISO and HACCP protocols are appropriately applied, then the danger is negligible. Such measures include not only the quality of raw materials and the hygiene status of the facilities but also the training and the professional behavior of the personnel during food handling. Personal hygiene, particularly hand washing and gloves wearing, are imperative not only in industrial scale production but also in small and medium scale businesses. If appropriately used in the food industry, gloves and masks can also reduce the spread of SARS-CoV-2 (180).

The usual cooking temperatures (>70°C) kill the virus, exerting a protective effect. In contrast, frozen food temperatures do not inactivate the virus making the possible transmission making, thus the washing of the hands a priority. Proper hand washing is an essential concept in food safety. FDA states that it can result in a 3-log reduction of bacteria and a 2-log reduction of viruses and protozoa (USFDA 2013, section 2-301.12) (182). Despite the emphasis given to proper hand washing, it often fails for various reasons such as lack of training, lack of means, time pressure and uncomfortable setting of relevant facilities (182). A common misunderstanding is that gloves can substitute hand washing. Using gloves can create a false sense of security, leading to unsafe practices such as less frequent and less proper hand washing (183, 184). All cleaning and antiseptic agents do not have the same power to eliminate viruses during hand washing. Antibacterial compounds in various antiseptics do not necessarily possess an antiviral effect. According to Sickbert-Bennett et al. (184), liquid detergents were 100 more potent in eliminating from hands respiratory viruses.

In a food-producing facility such as a restaurant, areas or points are classified as “high touch,” like taps, door handles, and refrigerator handles. These points should be cleaned and sanitized more frequently. The standard hygiene practices, which are applied to every food industry and can control foodborne bacteria and viruses, are also sufficient to hinder the dispersion of the SARS-CoV-2 virus. These practices include careful handling of raw materials to avoid cross-contamination, washing fruits and vegetables before serving, thoroughly cooking dishes and menus in temperatures above 70°C, and sneezing and coughing in the elbow (185–187).

The load of the viral RNA in urban sewage systems has been used as an epidemiological index to assess the infection status of a community. However, there are no studies available of the fate and the survival rate of the whole virus in the environment. This is a critical point because infected feces could become a source of environmental infection in developing countries where open defecation is commonly practiced. If the virus can survive even briefly, fresh vegetables and other fresh products could be infected through the irrigation system. Flies and other insects may carry the virus longer distances and infect surfaces, people and foods, particularly street foods. This kind of danger is enhanced in these countries by the lack of education in personal hygiene issues and by the shortage of means such as soap and antiseptics (188).

As the food supply chain moves from the farm or the field to the fork, the risk of viral contamination of the food or the packaging increases because more people are involved. The fact remains that the transmission of SARS CoV 2 through food has not been reported (189), and neither has been reported any case of orofecal transmission, with “so far” being the keyword. It is expected to be rare as an event but if the special conditions needed converge, then the orofecal transmission of the virus cannot be ruled out.

Revisiting the first period of the pandemic, it is now recognized that the most official lips, such as the World Health Organization and the American Food and Drug Association, ruled out the possibility of transmission of the virus through food, packaging materials and even though the staff of catering establishments in case they had fallen ill. The hazard was non-existent to infinitesimal, let alone if the virus infected one from a contaminated surface in a catering environment or the food industry (180, 190–192).

As early as 2020, a theory began to develop for an additional route of transmission of the coronavirus in addition to the already known ones. This particular route was about food storage and transportation, called the cold chain. 2 years later, this theory of virus transmission acquired a solid scientific background, documenting the survival of the virus in frozen food. The association of food imports with epidemic outbreaks in provinces of China were considered suspect. Indeed, it has been confirmed that the cold chain of food transport and storage can provide survival conditions for various coronas. The low and very low temperatures of multiple foods can harbor coronaviruses, e.g. SARS-CoV-1 (≈80% genome similarity to SARS-CoV-2), which according to the WHO, can be detected in frozen conditions (up to 2 years at −20°C), compared with their survival under normal temperatures (193–195). Foods considered substandard, tested by the health authorities of China, and confirmed as positive for the presence of SAS COVID-2 on their surfaces or packaging materials, were foods under refrigerated or frozen conditions such as ice cream, seafood, beer, pork, chicken, and fish (196).

## 4. Conclusions

This scoping review comprehensively addresses and provides valuable information on the crucial topic of hygiene standards and measures to combat the COVID-19 pandemic. Moreover, there is a compelling call for international and national public health bodies to delve into the relationship between outdoor and indoor air quality and its nexus with COVID-19's trajectory. It is expected that environmental factors and pandemic dynamics may be critically connected.

In addition, the review underscores the significance of delving into the interplay between COVID-19 and the food industry. Investigating food packaging materials and the virus's survival in food commodities could yield profound insights into global transmission patterns. Such research stands to enrich our understanding of viral dissemination across geographical borders.

In conclusion, this scoping review enriches our comprehension of hygiene measures in countering the pandemic. It underscores the necessity for interdisciplinary collaborations and targeted research to unravel the multifaceted dimensions of COVID-19's impact on various fronts.

## Data availability statement

The original contributions presented in the study are included in the article/Supplementary material, further inquiries can be directed to the corresponding author.

## Author contributions

CV, GR, ES, and EB: methodology, formal analysis, and writing—review & editing. CV, GR, and EB: software, validation, and visualization. CV, GR, ES, EG, CS, NV, CT, GV, YK, and EB: investigation. CV, GR, ES, CT, and EB: resources and writing—original draft preparation. VC, GR, EG, CS, NV, and EB: data curation. CV, GR, YK, and EB: supervision. CV and EB: project administration. All authors contributed to the article and approved the submitted version.

## References

[1] Van Regenmortel MHV Fauquet CM. Advances in viral toxonomy. Virologie. (2000) 4:1–5.

[2] Lodish H Berk A Zipursky SL Matsudaira P Baltimore PD. Viruses: Structure, Function, and Uses. In:Albert B, editor. Molecular Biology of the Cell 4th Edn. New York, NY: Garland Science. (2002).

[3] Decaro N Lorusso A. Novel human coronavirus (SARS-CoV-2): a lesson from animal coronaviruses. Vet Microbiol. (2022) 244:108693. 10.1016/j.vetmic.2020.108693 32402329 PMC7195271

[4] Munir K Ashraf S Munir I Khalid H Muneer MA Mukhtar N . Zoonotic and reverse zoonotic events of SARS-CoV-2 and their impact on global health. Emerg Microb Inf . (2020) 9:2222–35 10.1080/22221751.2020.1827984 32967592 PMC7594747

[5] World Health Organization (WHO). Coronavirus Disease (COVID-19): Vaccines. (2021). Available online at: https://www.who.int/emergencies/diseases/novel-coronavirus-2019/covid-19-vaccine

[6] World Health Organization (WHO). Interim Statement on Booster Doses for COVID-19 vaccination. (2021). Available online at: https://www.who.int/news/item/22-12-2021-interim-statement-on-booster-doses-for-covid-19-vaccination—update-22-december-2021 (accessed December 22, 2021).

[7] Cuicchi D Lazzarotto T Poggioli G. Fecal-oral transmission of SARS-CoV-2: review of laboratory-confirmed virus in gastrointestinal system. Int J Colorectal Dis. (2021) 36:437–44. 10.1007/s00384-020-03785-7 33057894 PMC7556558

[8] Amirian ES. Potential fecal transmission of SARS-CoV-2: current evidence and implications for public health. Int J Infect Dis. (2020) 95:363–70. 10.1016/j.ijid.2020.04.057 32335340 PMC7195510

[9] Gu J Han B Wang J. COVID-19: gastrointestinal manifestations and potential fecal–oral transmission. Gastroenterology. (2020) 158:1518–9. 10.1053/j.gastro.2020.02.054 32142785 PMC7130192

[10] Wang W Xu Y Gao R Lu R Han K Wu G . Detection of SARS-CoV-2 in different types of clinical specimens. JAMA. (2020) 323:1843–4. 10.1001/jama.2020.3786 32159775 PMC7066521

[11] Xiao F Tang M Zheng X Liu Y Li X Shan H. Evidence for gastrointestinal infection of SARS-CoV-2. Gastroenterology. (2020) 158:6. 10.1053/j.gastro.2020.02.055 32142773 PMC7130181

[12] Zhang Y Chen C Zhu S Shu C Wang D Song J . Isolation of 2019-nCoV from a stool specimen of a laboratory-confirmed case of the coronavirus disease 2019 (COVID-19). China CDC Wkly. (2020) 2:123–4. 10.46234/ccdcw2020.033 34594837 PMC8392928

[13] Jung SH Kim SW Lee H Oh JH Lim J. Serial screening for SARS-CoV-2 in rectal swabs of symptomatic COVID-19 patients. J Korean Med Sci. (2021) 36:e301. 10.3346/jkms.2021.36.e301 34783217 PMC8593410

[14] Lavania M Joshi MS Ranshing SS Potdar VA Shinde M Chavan N . Prolonged shedding of SARS-CoV-2 in Feces of COVID-19 positive patients: trends in genomic variation in first and second wave. Front Med. (2022) 9:835168. 10.3389/fmed.2022.835168 35372453 PMC8965355

[15] Wu Y Guo C Tang L Hong Z Zhou J Dong X . Prolonged presence of SARS-CoV-2 viral RNA in faecal samples. Lancet Gastroenterol Hepatol. (2020) 5:434–5. 10.1016/S2468-1253(20)30083-2 32199469 PMC7158584

[16] Joshi M Mohandas S Prasad S Shinde M Chavan N Yadav PD . Lack of evidence of viability and infectivity of SARS-CoV-2 in the fecal specimens of COVID-19 patients. Front Public Heal. (2022) 10:1030249. 10.3389/fpubh.2022.1030249 36339137 PMC9632423

[17] Cevik M Tate M Lloyd O Maraolo AE Schafers J Ho A. SARS-CoV-2, SARS-CoV, and MERS-CoV viral load dynamics, duration of viral shedding, and infectiousness: a systematic review and meta-analysis. The Lancet Microbe. (2021) 2:e13–22. 10.1016/S2666-5247(20)30172-5 33521734 PMC7837230

[18] Galbadage T Peterson BM Gunasekera RS. Does COVID-19 spread through droplets alone? Front Public Health. (2020) 24:163. 10.3389/fpubh.2020.00163 32391310 PMC7193306

[19] Water Sanitation Environmentally Related Hygiene (WASH). Centers for Disease Control and Prevention (CDC). (2022). Available online at: https://www.cdc.gov/hygiene/personal-hygiene/index.html#print (accessed December 1, 2022).

[20] Morawska L Cao J. Airborne transmission of SARS-CoV-2: The world should face the reality. Environ Int. (2020). 139:105730. 10.1016/j.envint.2020.105730 PMC7151430 32294574

[21] World Health Organization (WHO). Infection Prevention and Control During Health Care When Novel Coronavirus (nCoV) Infection is Suspected. Interim Guidance. (2020). Available online at: https://www.who.int/publications/i/item/WHO-2019-nCoV-HCF_assessment-IPC-2020.1

[22] World Health Organization (WHO). Responding to Community Spread of COVID-19. Interim Guidance. (2020). Available online at: https://www.who.int/publications/i/item/responding-to-community-spread-of-covid-19

[23] Güner R Hasanoglu I Aktaş F. COVID-19: prevention and control measures in community. Turk J Med Sci. (2020) 50:571–7. 10.3906/sag-2004-146 32293835 PMC7195988

[24] Huang F Armando M Dufau S Florea O Brouqui P Boudjema S. COVID-19 outbreak and healthcare worker behavioural change toward hand hygiene practices. J Hosp Infect. (2021) 111:27–34. 10.1016/j.jhin.2021.03.004 33716086 PMC7948529

[25] Stokel-Walker C. What do we know about covid vaccines and preventing transmission? BMJ. (2022) 376:298. 10.1136/bmj.o298 35121611

[26] Bonanno Ferraro G Veneri C Mancini P Iaconelli M Suffredini E Bonadonna L . State-of-the-art scoping review on SARS-CoV-2 in sewage focusing on the potential of wastewater surveillance for the monitoring of the COVID-19 pandemic. Food Environ Virol. (2022) 14:315–54. 10.1007/s12560-021-09498-6 34727334 PMC8561373

[27] Bartram J Hunter P. Bradley Classification of disease transmission routes for water-related hazards. In:Bartram J Baum R Coclanis PA and Gute DM, editors. Routledge Handbook of Water and Health. London: Routledge (2015).

[28] Patel KP Vunnam SR Patel PA Krill KL Korbitz PM Gallagher JP . Transmission of SARS-CoV-2: an update of current literature. Eur J Clin Microbiol Infect Dis. (2020) 39:2005–11. 10.1007/s10096-020-03961-1 32638221 PMC7339796

[29] Heneghan CJ Spencer EA Brassey J Plüddemann A Onakpoya IJ Evans DH . SARS-CoV-2 and the role of orofecal transmission: a systematic review. F1000Research. (2021) 10:231. 10.12688/f1000research.51592.2 35035883 PMC8749895

[30] Sucharew H Macaluso M. Methods for research evidence synthesis: the scoping review approach. J Hosp Med. (2019) 14:416–8. 10.12788/jhm.3248 31251164

[31] Munn Z Peters MDJ Stern C Tufanaru C McArthur A Aromataris E. Systematic review or scoping review? Guidance for authors when choosing between a systematic or scoping review approach. BMC Med Res Methodol. (2018) 18:11. 10.1186/s12874-018-0611-x 30453902 PMC6245623

[32] Gusenbauer M Haddaway NR. Which academic search systems are suitable for systematic reviews or meta-analyses? Res Syn Meth. (2020) 4:1–37. 10.1002/jrsm.1378 31614060 PMC7079055

[33] Falagas ME Pitsouni EI Malietzis G Pappas G. Comparison of PubMed, Scopus, Web of science, and google scholar: strengths and weaknesses. FASEB J. (2007) 22:338–42. 10.1096/fj.07-9492LSF 17884971

[34] Pal M Berhanu G Desalegn C Kandi V. Severe acute respiratory syndrome coronavirus-2 (SARS-CoV-2): an update. Cureus. (2020) 26:12. 10.7759/cureus.7423 PMC7182166 32337143

[35] Page MJ McKenzie JE Bossuyt PM Boutron I Hoffmann TC Mulrow CD . The PRISMA 2020 statement: an updated guideline for reporting systematic reviews. Int J Surg. (2021) 88:105906. 10.1016/j.ijsu.2021.105906 33789826

[36] Da T. Virology: coronaviruses. Nature. (1968) 220:650. 10.1038/220650b0

[37] Hierholzer JC Kemp MC Tannock GA. The RNA and proteins of human coronaviruses. Adv Exp Med Biol. (1981) 142:1–21. 10.1007/978-1-4757-0456-3_4 7337042

[38] Ziebuhr J. Preface. In:Dietzgen RG Francki RI, editors. Advances in Virus Research. London: Elsevier (2016). p. xiii–xiv. 10.1016/S0065-3527(16)30060-4 PMC7131292 27712629

[39] World Health Organization (WHO). COVID-19 Worldwide Dashboard - WHO Live World Statistics. Available online at: https://covid19.who.int/

[40] V'kovski P Kratzel A Steiner S Stalder H Thiel V. Coronavirus biology and replication: implications for SARS-CoV-2. Nat Rev Microbiol. (2021) 19:155–70. 10.1038/s41579-020-00468-6 33116300 PMC7592455

[41] Fehr AR Perlman S. Coronaviruses: an overview of their replication and pathogenesis. Coronaviruses: Methods Protoc. (2015) 25:1–23. 10.1007/978-1-4939-2438-7_1 25720466 PMC4369385

[42] Konda M Dodda B Konala VM Naramala S Adapa S. Potential zoonotic origins of SARS-CoV-2 and insights for preventing future pandemics through one health Approach. Cureus. (2020) 12:932. 10.7759/cureus.8932 32760632 PMC7392364

[43] Naqvi AAT Fatima K Mohammad T Fatima U Singh IK Singh A . Insights into SARS-CoV-2 genome, structure, evolution, pathogenesis and therapies: structural genomics approach. BBA Mol Basis Dis. (2020) 1866:165878. 10.1016/j.bbadis.2020.165878 32544429 PMC7293463

[44] Kaul D. An overview of coronaviruses including the SARS-2 coronavirus – Molecular biology, epidemiology and clinical implications. Curr Med Res Pract. (2020) 10:2. 10.1016/j.cmrp.2020.04.001 32363221 PMC7194867

[45] de Haan CAM te Lintelo E Li Z Raaben M Wurdinger T Bosch BJ . Cooperative Involvement of the S1 and S2 Subunits of the murine coronavirus spike protein in receptor binding and extended host range. J Virol. (2006) 80:22. 10.1128/JVI.00950-06 16956938 PMC1642182

[46] Huang Y Yang C Xu XF Xu W Liu SW. Structural and functional properties of SARS-CoV-2 spike protein: potential antivirus drug development for COVID-19. Acta Pharmacol Sin. (2020) 41:1141–9. 10.1038/s41401-020-0485-4 32747721 PMC7396720

[47] Lu R Zhao X Li J Niu P Yang B Wu H . Genomic characterisation and epidemiology of 2019 novel coronavirus: implications for virus origins and receptor binding. Lancet. (2020) 395:10224. 10.1016/S0140-6736(20)30251-8 32007145 PMC7159086

[48] Zhou P Yang X Lou Wang XG Hu B Zhang L Zhang W . Erratum: addendum: a pneumonia outbreak associated with a new coronavirus of probable bat origin. Nature. (1988) 588:81. 33199918 10.1038/s41586-020-2951-z PMC9744119

[49] Sallard E Halloy J Casane D Decroly E Van Helden J. Tracing the origins of SARS-COV-2 in coronavirus phylogenies: a review. Environ Chem Lett. (2021) 19:769–85. 10.1007/s10311-020-01151-1 33558807 PMC7859469

[50] Sironi M Hasnain SE Rosenthal B Phan T Luciani F Shaw MA . SARS-CoV-2 and COVID-19: a genetic, epidemiological, and evolutionary perspective. Inf Genetic Evol. (2020) 84:104384. 10.1016/j.meegid.2020.104384 32473976 PMC7256558

[51] Ni W Yang X Yang D Bao J Li R Xiao Y . Role of angiotensin-converting enzyme 2 (ACE2) in COVID-19. Critical Care. (2020) 24:1–10. 10.1186/s13054-020-03120-0 32660650 PMC7356137

[52] Gheblawi M Wang K Viveiros A Nguyen Q Zhong J-C Turner AJ . Angiotensin-converting enzyme 2: SARS-CoV-2 receptor and regulator of the renin-angiotensin system. Circ Res. (2020) 126:1–24. 10.1161/CIRCRESAHA.120.317015 PMC7188049 32264791

[53] Guo YR Cao QD Hong ZS Tan YY Chen SD Jin HJ . The origin, transmission and clinical therapies on coronavirus disease 2019 (COVID-19) outbreak–an update on the status. Military Med Res. (2020) 7:1–10. 10.1186/s40779-020-00240-0 32169119 PMC7068984

[54] Di Nardo M van Leeuwen G Loreti A Barbieri MA Guner Y Locatelli F . A literature review of 2019 novel coronavirus (SARS-CoV2) infection in neonates and children. Pediatr Res. (2021) 89:1101–8. 10.1038/s41390-020-1065-5 32679582

[55] Zhang H Penninger JM Li Y Zhong N Slutsky AS. Angiotensin-converting enzyme 2 (ACE2) as a SARS-CoV-2 receptor: molecular mechanisms and potential therapeutic target. Intensive Care Med. (2020) 46:4. 10.1007/s00134-020-05985-9 32125455 PMC7079879

[56] Shirbhate E Pandey J Patel VK Kamal M Jawaid T Gorain B . Understanding the role of ACE-2 receptor in pathogenesis of COVID-19 disease: a potential approach for therapeutic intervention. Pharmacol Rep. (2021) 73:1–12. 10.1007/s43440-021-00303-6 34176080 PMC8236094

[57] Ji W Wang W Zhao X Zai J Li X. Cross-species transmission of the newly identified coronavirus 2019-nCoV. J Med Virol. (2020) 92:433–40. 10.1002/jmv.25682 31967321 PMC7138088

[58] Liu Z Xiao X Wei X Li J Yang J Tan H . Composition and divergence of coronavirus spike proteins and host ACE2 receptors predict potential intermediate hosts of SARS-CoV-2. J Med Virol. (2020) 92:595–601. 10.1002/jmv.25726 32100877 PMC7228221

[59] Zhao J Cui W Tian BP. The potential intermediate hosts for SARS-CoV-2. Front Microbiol. (2020) 11:580137. 10.3389/fmicb.2020.580137 33101254 PMC7554366

[60] Xu R Cui B Duan X Zhang P Zhou X Yuan Q. Saliva: potential diagnostic value and transmission of 2019-nCoV. Int J Oral Sci. (2020) 12:11. 10.1038/s41368-020-0080-z 32300101 PMC7162686

[61] Kaufman E Lamster IB. The diagnostic applications of saliva—a review. Critic Rev Oral Biol Med. (2002) 13:197–212. 10.1177/154411130201300209 12097361

[62] To KKW Tsang OTY Yip CCY Chan KH Wu TC Chan JMC . Consistent detection of 2019 novel coronavirus in saliva. Clin Infect Dis. (2020) 71:841–3. 10.1093/cid/ciaa149 32047895 PMC7108139

[63] To KKW Tsang OTY Leung WS Tam AR Wu TC Lung DC . Temporal profiles of viral load in posterior oropharyngeal saliva samples and serum antibody responses during infection by SARS-CoV-2: an observational cohort study. Lancet Infect Dis. (2020) 20:565–74. 10.1016/S1473-3099(20)30196-1 32213337 PMC7158907

[64] Peng L Liu J Xu W Luo Q Deng K Lin B . 2019 Novel Coronavirus can be detected in urine, blood, anal swabs and oropharyngeal swabs samples. MedRxiv. (2020) 29:2020–02. 10.1101/2020.02.21.20026179 32330305 PMC7264521

[65] National Health Commission of the People's Republic of China. Notice on Issuing the Diagnosis and Treatment Plan for Pneumonia Infected by Novel Coronavirus (Trial Fourth Edition) (2020). Available online at: http://www.gov.cn/zhengce/zhengceku/2020-01/28/content_5472673.htm [Chinese].

[66] McIntosh K. COVID-19: Epidemiology, virology, and prevention. UpToDate^®^. (2019).

[67] World Health Organization (WHO). Transmission of SARS-CoV-2: Implications for Infection Prevention Precautions. Available online at: https://www.who.int/publications/i/item/modes-of-transmission-of-virus-causing-covid-19-implications-for-ipc-precaution-recommendations

[68] Burke RM Midgley CM Dratch A Fenstersheib M Haupt T Holshue M . Active monitoring of persons exposed to patients with confirmed COVID-19—United States, January–February 2020. Morbid Mortal Wkly Rep. (2020) 69:245. 10.15585/mmwr.mm6909e1 32134909 PMC7367094

[69] Burrell CJ Howard CR Murphy FA. Virion structure and composition. Fenner White's Med Virol. (2017) 27:27–37. 10.1016/B978-0-12-375156-0.00003-5

[70] Owusu D Pomeroy MA Lewis NM Wadhwa A Yousaf AR Whitaker B . Persistent SARS-CoV-2 RNA shedding without evidence of infectiousness: a cohort study of individuals with COVID-19. J Infect Dis. (2021) 224:1362–71. 10.1093/infdis/jiab107 33649773 PMC7989388

[71] Yilmaz A Marklund E Andersson M Nilsson S Andersson L-M Lindh M . Upper respiratory tract levels of severe acute respiratory syndrome coronavirus 2 RNA and duration of viral RNA shedding do not differ between patients with mild and severe/critical coronavirus disease 2019. J Infect Dis. (2021) 223:15–8. 10.1093/infdis/jiaa632 33020822 PMC7665561

[72] Bezirtzoglou E Stavropoulou E. Immunology and probiotic impact of the newborn and young children intestinal microflora. Anaerobe. (2011) 17:369–74. 10.1016/j.anaerobe.2011.03.010 21515397

[73] Stavropoulou E Bezirtzoglou E. Probiotics in medicine: a long debate. Front Immunol. (2020) 11:2192. 10.3389/fimmu.2020.02192 33072084 PMC7544950

[74] Yang B Fan J Huang J Guo E Fu Y Liu S . Clinical and molecular characteristics of COVID-19 patients with persistent SARS-CoV-2 infection. Nat Commun. (2021) 12:3501. 10.1038/s41467-021-23621-y 34108465 PMC8190301

[75] Fragkoudis R Dixon-Ballany C Zagrajek A Kedzierski L Fazakerley J. Following acute encephalitis, semliki forest virus is undetectable in the brain by infectivity assays but functional virus RNA capable of generating infectious virus persists for life. Viruses. (2018) 10:273. 10.3390/v10050273 29783708 PMC5977266

[76] Aiewsakun P Katzourakis A. Endogenous viruses: connecting recent and ancient viral evolution. Virology. (2015) 480:26–37. 10.1016/j.virol.2015.02.011 25771486

[77] Klenerman P Hengartner H Zinkernagel RM. A non-retroviral RNA virus persists in DNA form. Nature. (1997) 390:298–301. 10.1038/36876 9384383

[78] Griffin DE. Why does viral RNA sometimes persist after recovery from acute infections? PLoS Biol. (2022) 20:e3001687. 10.1371/journal.pbio.3001687 35648781 PMC9191737

[79] Cifuentes-Muñoz N Dutch RE Cattaneo R. Direct cell-to-cell transmission of respiratory viruses: the fast lanes. PLoS Pathog. (2018) 14:e1007015. 10.1371/journal.ppat.1007015 29953542 PMC6023113

[80] Wölfel R Corman VM Guggemos W Seilmaier M Zange S Müller MA . Virological assessment of hospitalized patients with COVID-2019. Nature. (2020) 588: 465–9. 10.1038/s41586-020-2196-x 32235945

[81] La Rochelle P Julien AS. How dramatic were the effects of handwashing on maternal mortality observed by Ignaz Semmelweis?. J Royal Soc Med. (2013) 106:459–60. 10.1177/0141076813507843 24158917 PMC3807775

[82] Jiehao C Jin X Daojiong L Zhi Y Lei X Zhenghai Q . A case series of children with 2019 novel coronavirus infection: clinical and epidemiological features. Clin Infect Dis. (2020) 71:1547–51. 10.1093/cid/ciaa198 32112072 PMC7108143

[83] Parasa S Desai M Chandrasekar VT Patel HK Kennedy KF Roesch T . Prevalence of gastrointestinal symptoms and fecal viral shedding in patients with coronavirus disease 2019: a systematic review and meta-analysis. JAMA Netw Open. (2020) 3:e2011335. 10.1001/jamanetworkopen.2020.11335 32525549 PMC7290409

[84] Zhang J Wang S Xue Y. Fecal specimen diagnosis 2019 novel coronavirus–infected pneumonia. J Med Virol. (2020) 92:680–2. 10.1002/jmv.25742 32124995 PMC7228355

[85] Xiao F Sun J Xu Y Li F Huang X Li H . Infectious SARS-CoV-2 in feces of patient with severe COVID-19. Emerg Infect Dis. (2020) 26:1920. 10.3201/eid2608.200681 32421494 PMC7392466

[86] Gwenzi W. Leaving no stone unturned in light of the COVID-19 faecal-oral hypothesis? A water, sanitation and hygiene (WASH) perspective targeting low-income countries. Sci Total Environ. (2021) 753:141751. 10.1016/j.scitotenv.2020.141751 32911161 PMC7438205

[87] Vivanti AJ Vauloup-Fellous C Prevot S Zupan V Suffee C Do Cao J . Transplacental transmission of SARS-CoV-2 infection. Nat Commun. (2020) 11:1–7. 10.1038/s41467-020-17436-6 32665677 PMC7360599

[88] Walker KF O'Donoghue K Grace N Dorling J Comeau JL Li W . Maternal transmission of SARS-CoV-2 to the neonate, and possible routes for such transmission: a systematic review and critical analysis. BJOG Int J Obstet Gynaecol. (2020) 127:1324–36. 10.1111/1471-0528.16362 PMC7323034 32531146

[89] Prabhu M Cagino K Matthews KC Friedlander RL Glynn SM Kubiak JM . Pregnancy and postpartum outcomes in a universally tested population for SARS-CoV-2 in New York City: a prospective cohort study. BJOG Int J Obstet Gynaecol. (2020) 127:1548–56. 10.1111/1471-0528.16403 32633022 PMC7361728

[90] World Health Organization (WHO). Handwashing an Effective Tool to Prevent COVID-19, Other Diseases. News Release SEARO. (2020). Available online at: https://www.who.int/southeastasia/news/detail/15-10-2020-handwashing-an-effective-tool-to-prevent-covid-19-other-diseases (accessed October 5, 2020).

[91] World Health Organization (WHO). Guidelines on Hand Hygiene in Health Care: First Global Patient Safety Challenge Clean Care is Safer Care. (2019). Available online at: http://apps.who.int/iris/bitstream/10665/44102/1/9789241597906_eng.pdf 23805438

[92] Israel S Harpaz K Radvogin E Schwartz C Gross I Mazeh H . Dramatically improved hand hygiene performance rates at time of coronavirus pandemic. Clin Microbiol Inf. (2020) 26:1566–8. 10.1016/j.cmi.2020.06.002 32526277 PMC7831641

[93] Sonigara BS Sarangdevot K Ranawat MS. Corona virus and soap: the supramolecular chemistry. Chem Res J. (2020) 2020:24–7.

[94] Suman R Javaid M Haleem A Vaishya R Bahl S Nandan D. Sustainability of coronavirus on different surfaces. J Clin Exp Hepatol. (2020) 10:386–90. 10.1016/j.jceh.2020.04.020 32377058 PMC7201236

[95] Rundle CW Presley CL Militello M Barber C Powell DL Jacob SE . Hand hygiene during COVID-19: recommendations from the American Contact Dermatitis Society. J Am Acad Dermatol. (2020) 83:1730–7. 10.1016/j.jaad.2020.07.057 32707253 PMC7373692

[96] Berardi A Perinelli DR Merchant HA Bisharat L Basheti IA Bonacucina G . Hand sanitisers amid CoViD-19: a critical review of alcohol-based products on the market and formulation approaches to respond to increasing demand. Int J Pharm. (2020) 584:119431. 10.1016/j.ijpharm.2020.119431 32461194 PMC7229736

[97] Anna A. Hand Sanitiser or Soap: Making an Informed Choice for COVID-19. Canberra: Australian Academy of Science. (2020).

[98] Ray I. Viewpoint–Handwashing and COVID-19: Simple, right there…? World Dev. (2020) 135:105086. 10.1016/j.worlddev.2020.105086 32834382 PMC7359802

[99] World Health Organization (WHO). Advice for the Public: Coronavirus Disease (COVID-19). Available online at: https://www.who.int/emergencies/diseases/novel-coronavirus-2019/advice-for-public

[100] Steere-Williams J. The Germ Theory. In:Montgomery GM Largent MA, editors. A Companion to the History of American Science. Chichester: John Wiley and Sons, Ltd (2015). p. 397–407.

[101] Tartari E Abbas M Pires D De Kraker MEA Pittet D. World health organization SAVE LIVES: clean your hands global campaign—‘fight antibiotic resistance—it's in your hands'. Clin Microbiol Inf. (2017) 23:596–8. 10.1016/j.cmi.2017.04.021 28487167

[102] Sax H Allegranzi B Uckay I Larson E Boyce J Pittet D. ‘My five moments for hand hygiene': a user-centred design approach to understand, train, monitor and report hand hygiene. J Hospital Inf. (2007) 67:9–21. 10.1016/j.jhin.2007.06.004 17719685

[103] European Centre for Disease Prevention and Control. Use of Gloves in Healthcare and Non-Healthcare Settings in the Context of the COVID 19 Pandemic. Stockholm: ECDC (2020).

[104] Best M Neuhauser D. Ignaz Semmelweis and the birth of infection control. BMJ Quality and Safety. (2004) 13:233–4. 10.1136/qshc.2004.010918 15175497 PMC1743827

[105] Artika IM Dewantari AK Wiyatno A. Molecular biology of coronaviruses: current knowledge. Heliyon. (2020) 6:e04743. 10.1016/j.heliyon.2020.e04743 32835122 PMC7430346

[106] Loudon I. Ignaz Phillip Semmelweis' studies of death in childbirth. J R Soc Med. (2013) 106:461–3. 10.1177/0141076813507844 24158918 PMC3807776

[107] Bischoff WE Reynolds TM Sessler CN Edmond MB Wenzel RP. Handwashing compliance by health care workers: the impact of introducing an accessible, alcohol-based hand antiseptic. Arch Intern Med. (2000) 160:1017–21. 10.1001/archinte.160.7.1017 10761968

[108] Trampuz A Widmer AF. Hand hygiene: a frequently missed lifesaving opportunity during patient care. In Mayo Clin Proc. (2004) 79:109–16. 10.4065/79.1.109 14708954 PMC7094481

[109] World Health Organization (WHO). Middle East Respiratory Syndrome Coronavirus (MERS-CoV). Geneva: WHO (2019).

[110] Korean Centers for Disease Control Prevention. Middle East Respiratory Syndrome. (2017). Available online at: http://www.cdc.go.kr/CDC/eng/contents/CdcEngContentView.jsp?cid=74219andmenuIds=HOME002-MNU0576-MNU0582

[111] Yang J Park EC Lee SA Lee SG. Associations between hand hygiene education and self-reported hand-washing behaviors among Korean adults during MERS-CoV outbreak. Health Educ Behav. (2019) 46:157–64. 10.1177/1090198118783829 30012018 PMC7207011

[112] Oh HS. Analysis of hand hygiene practices of health care personnels. J Korea Acad Ind Coop Soc. (2015) 16:6160–8. 10.5762/KAIS.2015.16.9.6160

[113] Yang NY Lee MS Hwang HJ Hong JY Kim BH Kim HS . Related factors of handwashing with soap and its practices by students in South Korea. J Korean Pub Health Nursing. (2014) 28:372–86. 10.5932/JKPHN.2014.28.2.372

[114] Lee YH Lee MS Hong S Yang NYHwang HJ Kim B-H. Related factors to handwashing with soap in Korean adults. J Korean Soc Sch Commun Heal Educ. (2016) 17:89–99.

[115] Lee M You M. Psychological and behavioral responses in South Korea during the early stages of coronavirus disease 2019 (COVID-19). Int J Environ Res Public Health. (2020) 17:2977. 10.3390/ijerph17092977 32344809 PMC7246607

[116] Tang H Lu X Su R Liang Z Mai X Liu C. Washing away your sins in the brain: physical cleaning and priming of cleaning recruit different brain networks after moral threat. Soc Cogn Affect Neurosci. (2017) 12:1149–58. 10.1093/scan/nsx036 28338887 PMC5490681

[117] Hermann C. Surprising Truth About Hand Hygiene During COVID-19. Jacksonville Beach, FL: Healthcare IT. (2021).

[118] Carducci A Federigi I Verani M. Covid-19 airborne transmission and its prevention: waiting for evidence or applying the precautionary principle? Atmosphere. (2020) 11:710. 10.3390/atmos11070710

[119] Wells WF. On air-borne infection. Study II Droplets and droplet nuclei. Am J Hygiene. (1934) 20:611–18. 10.1093/oxfordjournals.aje.a118097

[120] Santy-Tomlinson J. We need to talk about hand hygiene: a time to reflect on compliance. Int J Orthop Trauma Nursing. (2020) 39:100819. 10.1016/j.ijotn.2020.100819 32958424 PMC7471928

[121] Ebrahim SH Zhuo J Gozzer E Ahmed QA Imtiaz R Ahmed Y . All hands on deck: a synchronized whole-of-world approach for COVID-19 mitigation. Int J Inf Dis. (2020) 98:208–15. 10.1016/j.ijid.2020.06.049 32565364 PMC7301799

[122] Sahiledengle B Tekalegn Y Takele A Zenbaba D Teferu Z. Hand washing compliance and COVID-19. Prepr Serv Heal Sci. (2020) 14:1–2. 10.1101/2020.06.02.20120022

[123] Onyedibe KI Shehu NY Pires D Isa SE Okolo MO Gomerep SS . Assessment of hand hygiene facilities and staff compliance in a large tertiary health care facility in northern Nigeria: a cross sectional study. Antimicrob Resist Infect Control. (2020) 9:1–9. 10.1186/s13756-020-0693-1 32046790 PMC7014740

[124] Wong SC AuYeung CY Lam GM Leung EL Chan VM Yuen KY. Is it possible to achieve 100 percent hand hygiene compliance during the coronavirus disease 2019 (COVID-19) pandemic?. J Hos Inf. (2020) 105:779–81. 10.1016/j.jhin.2020.05.016 32422309 PMC7255117

[125] Głabska D Skolmowska D Guzek D. Population-based study of the influence of the COVID-19 pandemic on hand hygiene behaviors—Polish adolescents' COVID-19 experience (PLACE-19) study. Sustainability. (2020) 12:4930. 10.3390/su12124930

[126] Mardiko AA von Lengerke T. When, how, and how long do adults in Germany self-reportedly wash their hands? Compliance indices based on handwashing frequency, technique, and duration from a cross-sectional representative survey. Int J Hyg Environ Health. (2020) 230:113590. 10.1016/j.ijheh.2020.113590 32889358 PMC7462538

[127] Suen LKP So ZYY Yeung SKW Lo KYK Lam SC. Epidemiological investigation on hand hygiene knowledge and behaviour: a cross-sectional study on gender disparity. BMC Public Health. (2019) 19:401. 10.1186/s12889-019-6705-5 30975130 PMC6460727

[128] World Health Organization (WHO). Almost 2 Billion People Depend on Health Care Facilities Without Basic Water Services – WHO, UNICEF. (2020). Available online at: https://www.who.int/news/item/14-12-2020-almost-2-billion-people-depend-on-health-care-facilities-without-basic-water-services-who-unicef (accessed December 14, 2020).

[129] Sadule-Rios N Aguilera G. Nurses' perceptions of reasons for persistent low rates in hand hygiene compliance. Int Critic Care Nursing. (2017) 42:17–21. 10.1016/j.iccn.2017.02.005 28366521

[130] Erasmus V Daha TJ Brug H Richardus JH Behrendt MD Vos MC . Systematic review of studies on compliance with hand hygiene guidelines in hospital care. Inf Control Hosp Epidemiol. (2010) 31:283–94. 10.1086/650451 20088678

[131] Mahida N. Hand hygiene compliance: are we kidding ourselves? J Hosp Infect. (2016) 92:307–8. 10.1016/j.jhin.2016.02.004 26988123

[132] Lee SW Schwarz N. Washing away postdecisional dissonance. Science. (2010) 328:709–709. 10.1126/science.1186799 20448177

[133] World Health Organization (WHO). Infection Prevention and Control of Epidemic- and Pandemic-Prone Acute Respiratory Infections in Health Care. (2014). Available online at: https://apps.who.int/iris/bitstream/handle/10665/112656/9789241507134_eng.pdf?Sequence=1 24983124

[134] Moore LD Robbins G Quinn J Arbogast JW. The impact of COVID-19 pandemic on hand hygiene performance in hospitals. Am J Infect Control. (2021) 49:30–3. 10.1016/j.ajic.2020.08.021 32818577 PMC7434409

[135] Jayaweera M Perera H Gunawardana B Manatunge J. Transmission of COVID-19 virus by droplets and aerosols: a critical review on the unresolved dichotomy. Environ Res. (2020) 188:109819. 10.1016/j.envres.2020.109819 32569870 PMC7293495

[136] Fennelly KP. Particle sizes of infectious aerosols: implications for infection control. The Lancet Resp Med. (2020) 8:914–24. 10.1016/S2213-2600(20)30323-4 32717211 PMC7380927

[137] Judson SD Munster VJ. Nosocomial transmission of emerging viruses via aerosol-generating medical procedures. Viruses. (2019) 11:940. 10.3390/v11100940 31614743 PMC6832307

[138] Chen C Zhao B. Some questions on dispersion of human exhaled droplets in ventilation room: answers from numerical investigation. Indoor Air. (2010) 20:95–111. 10.1111/j.1600-0668.2009.00626.x 20002792

[139] Tellier R. Aerosol transmission of influenza A virus: a review of new studies. J the Royal Soc Interf. (2009) 6:S783–90. 10.1098/rsif.2009.0302.focus PMC2843947 19773292

[140] Balachandar S Zaleski S Soldati A Ahmadi G Bourouiba L. Host-to-host airborne transmission as a multiphase flow problem for science-based social distance guidelines. Int J Multiphase Flow. (2020) 132:103439. 10.1016/j.ijmultiphaseflow.2020.103439

[141] Shelton-Davenport M Pavlin J Saunders J Staudt A. Airborne Transmission of SARS-CoV-2. Washington, DC: National Academies Press (2020). 33119244

[142] Hsiao T-C Chuang H-C Griffith SM Chen S-J Young L-H. COVID-19: an aerosol's point of view from expiration to transmission to viral-mechanism. Aerosol Air Qual Res. (2020) 15:905–10. 10.4209/aaqr.2020.04.0154

[143] Tang S Mao Y Jones RM Tan Q Ji JS Li N . Aerosol transmission of SARS-CoV-2? Evidence, prevention and control. Environ Int. (2020) 144:106039. 10.1016/j.envint.2020.106039 32822927 PMC7413047

[144] Morawska L Tang JW Bahnfleth W Bluyssen PM Boerstra A Buonanno G Yao M. How can airborne transmission of COVID-19 indoors be minimised?. Environ Int. (2020) 142:105832. 10.1016/j.envint.2020.105832 32521345 PMC7250761

[145] Sagripanti JL Lytle CD. Estimated inactivation of coronaviruses by solar radiation with special reference to COVID-19. Photochem Photobiol. (2020) 96:731–7. 10.1111/php.13293 32502327 PMC7300806

[146] Riddell S Goldie S Hill A Eagles D Drew TW. The effect of temperature on persistence of SARS-CoV-2 on common surfaces. Virol J. (2020) 17:1–7. 10.1186/s12985-020-01418-7 33028356 PMC7538848

[147] Petersen E Koopmans M Go U Hamer DH Petrosillo N Castelli F . Comparing SARS-CoV-2 with SARS-CoV and influenza pandemics. Lancet Infect Dis. (2020) 20:e238–44. 10.1016/S1473-3099(20)30484-9 32628905 PMC7333991

[148] Dhand R Li J. Coughs and sneezes: their role in transmission of respiratory viral infections, including SARS-CoV-2. Am J Respir Crit Care Med. (2020) 202:651–9. 10.1164/rccm.202004-1263PP 32543913 PMC7462404

[149] Jarvis MC. Aerosol transmission of SARS-CoV-2: physical principles and implications. Front Public Heal. (2020) 8:590041. 10.3389/fpubh.2020.590041 33330334 PMC7719704

[150] National Academies of Sciences Engineering and Medicine. Rapid Expert Consultations on the COVID-19 Pandemic. Washington, DC: National Academies Press (US) (2020). 32407043

[151] Klompas M Baker MA Rhee C. Airborne transmission of SARS-CoV-2: theoretical considerations and available evidence. JAMA. (2020) 324:441–2. 10.1001/jama.2020.12458 32749495

[152] Correia G Rodrigues L Da Silva MG Gonçalves T. Airborne route and bad use of ventilation systems as non-negligible factors in SARS-CoV-2 transmission. Med Hypotheses. (2020) 141:109781. 10.1016/j.mehy.2020.109781 32361528 PMC7182754

[153] European Centre for Disease Prevention and Control. Heating, ventilation and air-conditioning systems in the context of COVID-19. Stockholm: ECDC (2020).

[154] Bhagat RK Wykes MD Dalziel SB Linden PF. Effects of ventilation on the indoor spread of COVID-19. J Fluid Mech. (2020) 903:F1. 10.1017/jfm.2020.720 34191877 PMC7520710

[155] Ahlawat A Wiedensohler A Mishra SK. An overview on the role of relative humidity in airborne transmission of SARS-CoV-2 in indoor environments. Aerosol Air Quality Res. (2020) 20:1856–61. 10.4209/aaqr.2020.06.0302

[156] Lewis D. Is the coronavirus airborne? Experts can't agree. Nature. (2020) 580:175. 10.1038/d41586-020-00974-w 32242113

[157] Noorimotlagh Z Jaafarzadeh N Martínez SS Mirzaee SA. A systematic review of possible airborne transmission of the COVID-19 virus (SARS-CoV-2) in the indoor air environment. Environ Res. (2021) 193:110612. 10.1016/j.envres.2020.110612 33309820 PMC7726526

[158] Harding H Broom A Broom J. Aerosol-generating procedures and infective risk to healthcare workers from SARS-CoV-2: the limits of the evidence. J Hosp Inf. (2020) 105:717–25. 10.1016/j.jhin.2020.05.037 32497651 PMC7263217

[159] World Health Organization (WHO). Advice on the Use of Masks in the Context of COVID-19. (2020). Available online at: https://www.who.int/publications/i/item/advice-on-the-use-of-masks-in-the-community-during-home-care-and-in-healthcare-settings-in-the-context-of-the-novel-coronavirus-(2019-ncov)-outbreak

[160] Birgand G Peiffer-Smadja N Fournier S Kerneis S Lescure FX Lucet JC. Assessment of air contamination by SARS-CoV-2 in hospital settings. JAMA network open. (2020) 3:e2033232–e2033232. 10.1001/jamanetworkopen.2020.33232 33355679 PMC7758808

[161] Nissen K Krambrich J Akaberi D Hoffman T Ling J Lundkvist Å . Long-distance airborne dispersal of SARS-CoV-2 in COVID-19 wards. Sci Rep. (2020) 10:19589. 10.1038/s41598-020-76442-2 33177563 PMC7659316

[162] Lednicky JA Lauzardo M Fan ZH Jutla A Tilly TB Gangwar M . Viable SARS-CoV-2 in the air of a hospital room with COVID-19 patients. Int J Inf Dise. (2020) 100:476–82. 10.1016/j.ijid.2020.09.025 32949774 PMC7493737

[163] Guo ZD Wang ZY Zhang SF Li X Li L Li C . Aerosol and surface distribution of severe acute respiratory syndrome coronavirus 2 in hospital wards, Wuhan, China, 2020. Emerg Infect Dis. (2020) 26:1586. 10.3201/eid2607.200885 32275497 PMC7323510

[164] Pollock AM Lancaster J. Asymptomatic transmission of covid-19. BMJ. (2020) 371. 10.1136/bmj.m4851

[165] Lee S Kim T Lee E Lee C Kim H Rhee H . Clinical course and molecular viral shedding among asymptomatic and symptomatic patients with SARS-CoV-2 infection in a community treatment center in the Republic of Korea. JAMA Intern Med. (2020) 180:1447–52. 10.1001/jamainternmed.2020.3862 32780793 PMC7411944

[166] Read R. A Choir Decided to go Ahead With Rehearsal. Now Dozens of Members Have COVID-19 two are Dead. Los Angeles Times. (2020). Available online at: https://www.latimes.com/world-nation/story/2020-03-29/coronavirus-choir-outbreak (accessed March 29, 2021).

[167] Somsen GA van Rijn C Kooij S Bem RA Bonn D. Small droplet aerosols in poorly ventilated spaces and SARS-CoV-2 transmission. Lancet Respir Med. (2020) 8:658–9. 10.1016/S2213-2600(20)30245-9 32473123 PMC7255254

[168] Eames I Tang JW Li Y Wilson P. Airborne transmission of disease in hospitals. J the Royal Soc Interf. (2009) 6:S697–702. 10.1098/rsif.2009.0407.focus 19828499 PMC2843953

[169] Nishiura H Oshitani H Kobayashi T Saito T Sunagawa T Matsui T Suzuki M. Closed environments facilitate secondary transmission of coronavirus disease 2019 (COVID-19). MedRxiv. (2020) 37:1–4. 10.1101/2020.02.28.20029272

[170] Miller SL Clements N Elliott SA Subhash SS Eagan A Radonovich LJ. Implementing a negative-pressure isolation ward for a surge in airborne infectious patients. Am J Infect Control. (2017) 45:652–9. 10.1016/j.ajic.2017.01.029 28330710 PMC7115276

[171] CEN Standard. EN 16798-7:2017. Energy Performance of Buildings – Ventilation for Buildings. CEN Standard EN 16798–7: 2017. Part 7: Calculation Methods for the Determination of Air Flow Rates in Buildings Including Infiltration. European Committee for Standardization. (2017). Available online at: https://standards.iteh.ai/catalog/standards/cen/fe7f3c3d-8b66-4c06-a89b-643902c86545/en-16798-7-2017

[172] ANSI/ASHRAE. ANSI/ASHRAE Standard 52.2-2017, Method of Testing General Ventilation Air-Cleaning Devices for Removal Efficiency by Particle Size. (2017). Available online at: https://www.ashrae.org/FileLibrary/Technical~Resources/COVID-19/52_2_2017_COVID-19_20200401.pdf

[173] Jefferson T Spencer EA Brassey J Heneghan C. SARS-CoV-2 and the role of orofecal transmission: evidence brief. Public Health. (2020) 95:363–70.35035883

[174] Zhang H Li HB Lyu JR Lei XM Li W Wu G . Specific ACE2 expression in small intestinal enterocytes may cause gastrointestinal symptoms and injury after 2019-nCoV infection. Int J Inf Dis. (2020) 96:19–24. 10.1016/j.ijid.2020.04.027 32311451 PMC7165079

[175] Lamers MM Beumer J van der Vaart J Knoops K Puschhof J Breugem TI . SARS-CoV-2 productively infects human gut enterocytes. Science. (2020) 369:50–4. 10.1126/science.abc1669 32358202 PMC7199907

[176] Gupta A Madhavan MV Sehgal K Nair N Mahajan S Sehrawat TS . Extrapulmonary manifestations of COVID-19. Nat Med. (2020) 26:1017–32. 10.1038/s41591-020-0968-3 32651579 PMC11972613

[177] Young BE Ong SWX Kalimuddin S Low JG Tan SY Loh J. Epidemiologic features and clinical course of patients infected with SARS-CoV-2 in Singapore. JAMA. (2020) 323:1488–94. 10.1001/jama.2020.3204 32125362 PMC7054855

[178] Moura IB Buckley AM Wilcox MH. Can SARS-CoV-2 be transmitted via faeces?. Curr Opin Gastroenterol. (2022) 38:26. 10.1097/MOG.0000000000000794 34628417 PMC8654121

[179] Van Doremalen N Bushmaker T Morris DH Holbrook MG Gamble A Williamson BN . Aerosol and surface stability of SARS-CoV-2 as compared with SARS-CoV-1. New Eng J Med. (2020) 382:1564–7. 10.1056/NEJMc2004973 32182409 PMC7121658

[180] World Health Organization (WHO) / Food Agriculture Organization (FAO). COVID-19 and Food Safety: Guidance for Food Businesses: Interim Guidance 7 April 2020, WHO Ref. Number WHO/2019-NCoV/Food_Safety/2020.1. (2020). Available online at: https://apps.who.int/iris/bitstream/handle/10665/331705/WHO-2019-nCoV-Food_Safety-2020.1-eng.pdf (accessed February 21, 2023).

[181] Food Code 2013. U.S. Public Health Service, Food Drug Administration. (2013). Available online at: https://www.fda.gov/media/87140/download

[182] Food Code 2013. United States Public Health Service. Food Drug Administration. (2013). Available online at: https://www.fda.gov/media/87140/download

[183] Green L Selman C Banerjee A Marcus R Medus C Angulo FJ . Food service workers' self-reported food preparation practices: an EHS-Net study. Int J Hyg Environ Health. (2005) 208:27–35. 10.1016/j.ijheh.2005.01.005 15881976

[184] Sickbert-Bennett EE Weber DJ Gergen-Teague MF Sobsey MD Samsa GP Rutala WA. Comparative efficacy of hand hygiene agents in the reduction of bacteria and viruses. Am J Infect Control. (2005) 33:67–77. 10.1016/j.ajic.2004.08.005 15761405 PMC7252025

[185] Rizou M Galanakis IM Aldawoud TM Galanakis CM. Safety of foods, food supply chain and environment within the COVID-19 pandemic. Trends Food Sci Technol. (2020) 102:293–9. 10.1016/j.tifs.2020.06.008 32834502 PMC7295520

[186] Safefood. COVID-19 Advice - Safe Food. (2020). Available online at: https://www.safefood.qld.gov.au/covid-19-advice

[187] Seymour N Yavelak M. CCCB COVID-19 and food safety FAQ: Is coronavirus a Concern With Takeout? IFAS Extension. Gainesville, FL: University of Florida (2020).

[188] Saba CKS. COVID-19: implications for food, water, hygiene, sanitation, and environmental safety in Africa: a case study in Ghana. Preprints 2020, 2020050369. 10.20944/preprints202005.0369.v1

[189] FAO. COVID-19: Guidance for Preventing Transmission of COVID-19 within Food Businesses. Updated Guidance. Rome (2021). 10.4060/cb6030en

[190] World Health Organization (WHO). COVID-19, Virtual Press Conference, 13 August 2020. Geneva: WHO (2020).

[191] Centers for Disease Control Prevention (CDC). Food and Coronavirus Disease 2019 (COVID-19). (2020). Available online at: https://stacks.cdc.gov/view/cdc/89690/cdc_89690_DS1.pdf (accessed February 23, 2023).

[192] Centers for Disease Control Prevention (CDC). How COVID-19 Spreads. (2020). Available online at: https://www.cdc.gov/coronavirus/2019-ncov/prevent-getting-sick/how-covid-spreads.html (accessed February 21, 2023).

[193] Han J Zhang X He S Jia P. Can the coronavirus disease be transmitted from food? A review of evidence, risks, policies and knowledge gaps. Environ Chem Lett. (2021) 19:5–16. 10.1007/s10311-020-01101-x 33024427 PMC7529092

[194] World Health Organization (WHO). Coronavirus Disease 2019 (COVID-19) Situation Report 32. (2020). Available online at: https://www.who.int/docs/default-source/coronaviruse/situation-reports/20200221-sitrep-32-COVID-19.pdf?sfvrsn=4802d089_2

[195] Blondin-Brosseau M Harlow J Doctor T Nasheri N. Examining the persistence of human Coronavirus 229E on fresh produce. Food Microbiol. (2021). 98:103780. 10.1016/j.fm.2021.103780 33875208 PMC7909902

[196] Dai H Tang H Sun W Deng S Han J. It is time to acknowledge coronavirus transmission via frozen and chilled foods: undeniable evidence from China and lessons for the world. Sci Total Environ. (2023) 868:161388. 10.1016/j.scitotenv.2023.161388 36621479 PMC9814272

